# 2023 ACVIM Forum Research Report Program

**DOI:** 10.1111/jvim.16902

**Published:** 2023-11-06

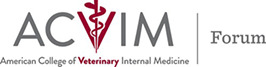

The American College of Veterinary Internal Medicine (ACVIM) Forum and the Journal of Veterinary Internal Medicine (JVIM) are not responsible for the content or dosage recommendations in the abstracts. The abstracts are not peer reviewed before publication. The opinions expressed in the abstracts are those of the author(s) and may not represent the views or position of the ACVIM. The authors are solely responsible for the content of the abstracts.

**2023 ACVIM Forum**

**June 14 to October 31, 2023**

**Research Report Program**

**Index of Abstracts**

**Table:** 

**THURSDAY, JUNE 15**		
Time	Presenting Author	Abstract Title
**EQUINE**		
4:30 pm	Shune Kimura	Insulin and Metformin Modulate Equine Neutrophil ROS Production Ex Vivo
5:00 pm	Linda Paul	Bile Acid Profiling of Pyloric Gastric Fluid in Horses with Equine Glandular Gastric Disease
5:30 PM	Breanna Sheahan	Successful Establishment of Equine Duodenal Organoids From Endoscopically‐Guided Biopsies
**NEUROLOGY**		
3:00 PM	Patrick Roynard	Surgical Experience with Lumbosacral (Myelo)meningoceles with Tethered Cord Syndrome (TCS) in 8 Bulldog Puppies
3:30 PM	Jihey Lim	Circulating microRNA Profiles in Dogs with Acute Spinal Cord Injury
**SMALL ANIMAL INTERNAL MEDICINE**		
10:00 am	Kayla Dunn	Effects of Preservative and Volume on Diagnostic Parameters for Urine Samples From Dogs and Cats
10:30 am	JD Foster	Use of Sodium Zirconium Cyclosilicate for the Management of Hyperkalemia in Dogs and Cats
11:15 am	Jessica Quimby	The Effect of Gabapentin on Blood Pressure in Cats with and without Chronic Kidney Disease
11:45 am	Dennis Woerde	Evaluation of Serum Galectin‐3 Concentrations in Healthy Cats and in Cats with Ureteral Obstruction
**FRIDAY, JUNE 16**		
Time	Presenting Author	Abstract Title
**CARDIOLOGY**		
1:30 PM	Alondramaria Garcia‐Revilla	Identification of Novel MicroRNAs in the Heart from Cats with Hypertrophic Cardiomyopathy
2:00 PM	Joshua Stern	Oral Rapamycin Therapy in Feline Subclinical Hypertrophic Cardiomyopathy: Results of the RAPACAT Clinical Trial
2:45 pm	Sonya Wesselowski	Electrocardiographic Findings in Clinical Healthy Adult Borzoi Dogs with a Normal Echocardiogram
3:15 pm	Lauren Markovic	Balloon Valvuloplasty for Pulmonary Valve Stenosis: Multicenter Collaborative Study Across Pediatric and Veterinary Cardiology Centers
4:30 PM	Noriko Isayma	Four Techniques for the Standardization of Canine Mitral Valve Repair
5:00 PM	Brianna Potter	Feasibility Clinical Study of Transcatheter Edge‐to‐Edge Repair in Dogs with the Canine Mitral V‐Clamp Device
5:30 PM	Kevin Phipps	Left Atrial Decompression in Dogs with Mitral Valve Disease and Heart Failure: Long Term Outcome
**EQUINE**		
8:00 AM	Nicholas Bamford	Effect of Thyrotropin Releasing Hormone and Pergolide on Plasma β‐Endorphin Concentrations in Horses and Ponies
8:30 AM	Nicholas Frank	Effects of the Sodium‐Glucose Cotransporter‐2 Inhibitor Velagliflozin on Insulin Concentrations in Horses With Insulin Dysregulation
9:15 AM	Marion Allano	Staphylococcus aureus Nasal Colonization and Risk Factors in Horses Admitted to A Veterinary Teaching Hospital
9:45 AM	Emma Gorenberg	Xylazine and Morphine Caudal Epidural Analgesia in Horses: Influence of Volume on Location of Effect
**FOOD ANIMAL**		
11:00 AM	Joe Smith	The Pharmacokinetics and Efficacy of Intravenous Single Dose Esomeprazole in Sheep
11:30 AM	Joe Smith	Pharmacokinetics and Efficacy of Pantoprazole in Sheep After Intravenous and Subcutaneous Administration
5:30 PM	Amelia Woolums	Tulathromycin Metaphylaxis Increases Isolation of Multidrug Resistant Mannheimia haemolytica in Stocker Heifers
**NUTRITION**		
11:00 AM	Valerie Parker	Ionized Hypercalcemia Resolves with Nutritional Modification in Cats with Idiopathic Hypercalcemia and Chronic Kidney Disease
11:30 AM	Aulus Carciofi	Starch:Protein Ratio in Energy Expenditure and Body Composition in Neutered and Intact Male Cats
**ONCOLOGY**		
1:30 PM	Jenna Burton	Frequency of Cancer Following Screening in Higher Risk, Middle‐Aged to Older Dogs
2:00 PM	Amy LeBlanc	The Integrated Canine Data Commons (ICDC): Two Year Experience Report
**SMALL ANIMAL INTERNAL MEDICINE**		
8:00 AM	Ellen Behrend	Velagliflozin, an SGLT2 Inhibitor, as Once‐Daily, Oral Solution, Stand‐Alone Therapy for Feline Diabetes Mellitus
8:30 AM	J. Catharine Scott‐Moncrieff	Treatment with the Sodium‐Glucose Cotransporter‐2 Inhibitor Bexagliflozin in Cats Newly Diagnosed with Diabetes Mellitus
9:15 AM	Lisa Stammeleer	Blood Pressure in Hyperthyroid Cats Before and After Radioiodine Treatment
9:45 AM	Jennifer Reinhart	Itraconazole Metabolite Biotransformation by Hepatic Cytochrome P450 Enzymes in the Dog
11:00 AM	Kaitlin Daly	Characterizing the Intrinsic Nervous System of Gallbladders from Normal Dogs and Dogs with Mucocele Formation
11:30 AM	Alyssa Berman	Correlation Between Urine Anion Gap and Urine Ammonia‐Creatinine Ratio in Healthy and Kidney Disease Cats
1:30 PM	Anne Avery	Evaluation of Histology, Immunohistochemistry, Clonality and STAT5B Mutation Detection in Cats with Chronic Enteropathy
2:00 PM	Patrick Barko	Microbial Indole Catabolites of Tryptophan in Cats with Chronic Enteropathy
2:45 PM	Alison Manchester	Single Cell RNA Sequencing Atlas of Canine Duodenal Mucosa in Chronic Inflammatory Enteropathy and Health
3:15 PM	Tarini Ullal	Evaluating Acidic Gastroesophageal Reflux in French Bulldogs with Sliding Hiatal Herniation Using Bravo pH Monitoring
4:30 PM	Jared Jaffey	Tracheobronchial Lymphadenopathy in Dogs with Pulmonary Coccidioidomycosis
5:00 PM	Christian Leutenegger	Association of the Novel Benzimidazole Resistance Marker Q134H with F167Y in Dogs with Ancylostoma caninum
5:30 PM	Laura Van Vertloo	Gastrointestinal Adverse Effects of Ophthalmic Nonsteroidal Anti‐inflammatory Drugs in Dogs

## CARDIOLOGY

## Identification of novel microRNAS in the heart from cats with hypertrophic cardiomyopathy

### **Alondramaria** **Garcia‐Revilla** ^1^ ; Jessica Joshua^1^; Sonja Fonfara^2^

#### ^1^University of Guelph, Guelph, ON, USA; ^2^Ontario Veterinary College, University of Guelph, Guelph, ON, USA

**Background:** Hypertrophic cardiomyopathy (HCM) is the most common feline heart disease; its pathogenesis is largely unknown. MicroRNAs have been reported to play a role in human HCM pathogenesis. MicroRNA sequencing of feline HCM hearts was carried out, which identified known and novel microRNAs.

**Objective**: Evaluate novel microRNAs identified in hearts from cats with HCM.

**Animals**: Five left ventricles (LV) and 5 left atria (LA) from cats with HCM (group 1), and 7 LV and 5 LA from healthy cats (group 2).

**Methods**: MicroRNAs from the LV and LA of group 1 and 2 were compared using miRDeep2. Novel microRNA sequences were evaluated to determine homologs (BLAST) and to assess their candidacy (RNAfold).

**Results**: Comparing the LV with the LA in each group identified 33 differentially expressed novel microRNAs in group 1 and 46 in group 2. Fifteen were found in both the HCM and healthy LV, indicating chamber specificity. Fifty‐five and 44 novel microRNAs were differently expressed comparing the LV and LA respectively from group 1 with group 2. Forty‐four of the LV and 33 of the LA microRNAs were chamber specific. Eleven microRNAs (7 down‐regulated, 4 up‐regulated) were similarly expressed in both the LV and LA, suggesting chamber‐independent involvement in HCM. Thirty microRNAs were homologs, 29 were orthologs, and for 29 microRNAs no species overlap was identified, indicating the discovery of microRNAs unreported so far.

**Conclusions**: This study identified HCM and cardiac‐chamber specific novel microRNAs. Further investigations into their role in feline HCM pathogenesis is required.

## Four techniques for the standardization of canine mitral valve repair

### **Noriko Isayama** ^1^; Sayaka Suzuki^2^, DVM; Kenta Sasaki^3^, DVM; Erika Maeda^3^, DVM; Yusuke Uchimura^4^, DVM; Takeshi Mizuno^5^, DVM, PhD

#### ^1^Tokyo Animal CardioThoracic Surgery (TACTS), Uenonomori Animal Hospital, Tokyo, Japan; ^2^Japan Small Animal Medical Center, Tokyo, Japan; ^3^Uenonomori Animal Hospital, Tokyo, Japan; ^4^President, Uenonomori Animal Hospital, Tokyo, Japan; ^5^Graduate School of Agricultural and Life Sciences, The University of Tokyo, Tokyo, Japan

**Background**: The surgical options for treating mitral insufficiency (MI) in dogs are influenced by multiple factors, including the surgeon's expertise and proficiency. Therefore, surgical management of this disease is not standardized.

**Hypothesis/Objectives**: This study established a standardized technique for controlling regurgitation, independent of the surgeon's experience.

**Animals**: The study included 19 client‐owned dogs with MI, treated surgically via the key points technique. Surgery was performed by 1 of 2 surgeons with different experience levels.

**Methods**: The surgical technique for MI was standardized using 4 key points (MI‐4):

1. Determining the valve annular dimension by measuring the anterior leaflet
2. Utilizing the Triad Anchored chordae tendineae reconstruction method
3. Determining the appropriate position and number of chordae tendineae on the anterior and posterior leaflets
4. Determining the appropriate height

The percentage of regurgitation (%Reg) was measured using the B‐mode color Doppler flow of the atrium on the four‐chamber left long‐axis view.

**Results**: There were 10 and 9 dogs with stage C and D disease, respectively. All 19 dogs were discharged, and 18 dogs were successfully reevaluated after 1 month. The %Reg decreased from 73% (interquartile range [IQR], 59.6%‐79.3%) to 4.3% (IQR, 0.2%‐11.5%) All dogs showed clinical improvement.

**Conclusions and Clinical Importance**: The MI‐4 surgical technique reduced the number of intraoperative decision points, eliminated dependence on the surgeon's experience, effectively controlled regurgitation, and prevented intraoperative complications. Additionally, the results could guide future research and aid veterinary surgeons in making more informed decisions when treating dogs with MI.

## Balloon valvuloplasty for pulmonary valve stenosis: Multicenter collaborative study across pediatric and veterinary cardiology centers

### **Lauren E. Markovic** ^1^; Brian Scansen^2^, DVM, MS, DACVIM (Cardiology); Gurumurthy Hiremath^3^, MD, FACC, FSCAI, FPICS; Heidi Kellihan^4^, DVM, DACVIM (Cardiology); Amanda Coleman^5^, DVM, DACVIM (Cardiology); Sonja Tjostheim^4^, DVM, DACVIM (Cardiology); Caitlin Calkins^6^, NP; Katie Hodges^7^, RPhT; Erin Cahill^8^; Brianne Tainter^9^, PA‐C; Mary Carter^10^, DVM; Dennis Kim^11^, MD, PhD, FSCAI

#### ^1^College of Veterinary Medicine, University of Georgia, Athens, GA, USA; ^2^Professor, Colorado State University, Fort Collins, CO, USA; ^3^Associate Professor, Masonic Children's Hospital, University of Minnesota, Minneapolis, MN, USA; ^4^Clinical Associate Professor, University of Wisconsin; ^5^Associate Professor, University of Georgia, Athens, GA, USA; ^6^Pediatric Nurse Practitioner, Cardiac Cath Lab, Children's Healthcare of Atlanta, Atlanta, GA, USA; ^7^Research Technician, University of Georgia, Athens, GA, USA; ^8^DVM Student, Colorado State University, Fort Collins, CO, USA; ^9^Physician Assistant, Masonic Children's Hospital, University of Minnesota, Minneapolis, MN, USA; ^10^University of Wisconsin; ^11^Professor, Children's Healthcare of Atlanta, School of Medicine, Emory University, Atlanta, GA, USA

**Background**: Transcatheter therapeutics have revolutionized treatment of pulmonary valve stenosis (PS) in children and animals. Further understanding of PS in humans and animals may help improve outcomes for both populations.

**Hypothesis/Objectives**: To describe characteristics and outcomes of PS in patients that underwent balloon pulmonary valvuloplasty (BPV) in pediatric and veterinary populations.

**Animals**: Seventy‐eight human patients from 2 pediatric cardiology centers, and 165 dogs from 3 veterinary cardiology centers.

**Methods**: Multicenter, retrospective review analyzing PS patients between July 1, 2019 and June 30, 2021. Demographics, procedural characteristics, and outcomes were assessed. Data are presented as mean ± SD.

**Results**: The stenosis was valvar in the majority of patients, 64/78 (82%) human and 141/165 (86%) dogs, with no difference between groups (*P* = .50). Initial echocardiographic peak transpulmonary pressure gradients were significantly higher in dogs (canine 122 mm Hg ±39; human 70 mm Hg ±22; *P* < .0001). Dogs were more likely to be receiving a beta blocker at intervention, 151/165 (92%), compared to humans 2/78 (3%) (*P* < .0001). Proportion of humans with congestive heart failure at diagnosis was 0/78, compared to 14/164 (9%) dogs (*P* = .006). The balloon: annulus ratio was significantly different (canine 1.28 ± 0.24; human 1.19 ± 0.3) (*P* < .0001). BPV was successful, based on a 50% or more reduction in transpulmonary pressure gradient, in 139/165 (84%) dogs and 75/78 (96%) humans (*P* = .008).

**Conclusions**: Humans and dogs with PS have similar morphology and response to BPV. Pre‐procedural transpulmonary pressure gradients are higher in dogs compared to humans. Beta blockers are more commonly prescribed to dogs than humans.

## Left atrial decompression in dogs with mitral valve disease and heart failure: Long term outcome

### **Kevin L. Phipps**, DACVIM (Cardiology); Justin Allen, DACVIM (Cardiology); Kirstie Barrett, DACVIM (Cardiology)

#### VCA West Los Angeles Animal Hospital, Los Angeles, CA, USA

**Background**: Left‐sided congestive heart failure (CHF) secondary to myxomatous mitral valve disease (MMVD) is associated with significant morbidity and mortality in the canine patient. Left atrial decompression (LAD) has been used to treat severely affected dogs who are failing standard medical therapy for a variety of reasons. No data has been published regarding the long‐term outcome of dogs who have undergone LAD.

**Hypothesis/Objectives**: The objective of our study was to determine incidence of atrial septal defect closure, frequency of right‐sided congestive heart failure, frequency of hospitalizations after LAD, and survival times.

**Animals**: A total of 111 client‐owned dogs who were afflicted with ACVIM stage B2 (late), C, or D MMVD and underwent LAD.

**Methods**: Retrospective cohort study enrolling patients with MMVD who had LAD performed between October 2018 and September 2021. Primary outcome variable was time to cardiac‐related death or euthanasia. Secondary variables were time to right‐sided CHF, artrial spetal defect closure, and number of hospitalizations for CHF post‐LAD.

**Results**: Closure of the iatrogenic atrial septal defect occurred in 22 dogs (20%). Iatrogenic atrial septal defect closure occurred most commonly in defects that were located caudally to the fossa ovalis. Forty dogs (36%) developed right‐sided CHF as a sequela to the LAD, which occurred between 1 and 800 days post‐operatively. 25/111 (22.5%) patients had hospitalizations within their lifetime post‐LAD as a result of cardiac disease. Median survival time post‐LAD was 379 days with a range of 1 to 1282 days.

**Conclusions and Clinical Importance**: Left atrial decompression was associated with a satisfactory survival time, given the severity of disease in the patient population. Right‐sided congestive heart failure is common after the procedure, though onset is variable. Closure of the iatrogenic atrial septal defect is uncommon, and incidence can be minimized by ensuring transseptal puncture occurs within the fossa ovalis.

## Feasibility clinical study of transcatheter edge‐to‐edge repair in dogs with the canine mitral V‐Clamp device

### **Brianna M. Potter** ^1^; E. Christopher Orton^2^, DVM, PhD, DACVS; I‐Jung Chi^3^, DVM, MS; Brian Scansen^2^, DVM, MS, DACVIM (Cardiology); Katie Abbott‐Johnson^1^ , DVM, MS; Lance Visser^4^, DVM, MS, DACVIM (Cardiology); Ellen Shaub^5^, AS

#### ^1^Veterinary Teaching Hospital, Colorado State University, Fort Collins, CO, USA; ^2^Professor, Cardiology and Cardiac Surgery, Veterinary Teaching Hospital, Colorado State University, Fort Collins, CO, USA; ^3^Cardiology Resident, Cardiology and Cardiac Surgery, Veterinary Teaching Hospital, Colorado State University, Fort Collins, CO, USA; ^4^Associate Professor, Cardiology and Cardiac Surgery, Veterinary Teaching Hospital, Colorado State University, Fort Collins, CO, USA; ^5^Anesthesia, Veterinary Teaching Hospital, Colorado State University, Fort Collins, CO, USA

**Background**: Transcatheter edge‐to‐edge mitral valve repair (TEER) has been shown in pivotal studies to be an effective treatment for degenerative mitral regurgitation (MR) in humans.

**Hypothesis/Objective**: Determine feasibility, acute safety, adverse device‐related events, early efficacy, and freedom from all‐cause and cardiac‐related mortality of a canine‐specific TEER device (V‐Clamp) in dogs with degenerative MR.

**Animals**: Initial inclusion criteria were dogs with stage C or D degenerative MR. During the study, stage B2 dogs were added if they met guidelines for severe MR.

**Methods**: Prospective single‐arm single‐institution FDA feasibility‐type study. TEER was performed via a transapical approach under transesophageal echocardiography and fluoroscopic guidance.

**Results**: Forty dogs were enrolled over a 2‐year period (12 B2, 26 C, 2 D). Procedural feasibility was 95% based on successful delivery of at least one clamp in 38/40 dogs. There were no procedural deaths. Acute procedural safety was 95% based on survival to hospital discharge of 38/40 dogs. Most dogs were discharged by the 2nd day. Adverse device‐related event rate was 6.2% based on 3 events (1 single‐leaflet detachment, 1 clamp unlock, 1 clamp embolization) in 48 implanted clamps. All 3 events were nonfatal and successfully treated with a second clamp. Regurgitant fraction and volume decreased from 64% ± 12% and 2.4 ± 0.9 mL/kg at baseline to 43 ± 26% (*P* < .0001) and 1.3 ± 1.1 mL/kg (*P* < .0001) at hospital discharge. Freedom from all‐cause and cardiac‐related mortality at 9 months was 87.4% and 91.1%, respectively.

**Conclusions**: Initial feasibility results support continued development of TEER as a low‐risk and effective treatment for degenerative MR in dogs.

## Electrocardiographic findings in clinical healthy adult Borzoi dogs with a normal echocardiogram

### **Sonya R. Wesselowski** ^1^; Blakeley Janacek^2^, DVM, DACVIM (Cardiology); K. Tess Sykes^1^, DVM; Ashley Saunders^3^, DVM, DACVIM (Cardiology)

#### ^1^Texas A&M University, College Station, TX, USA; ^2^Cardiologist, VCA Animal Diagnostic Clinic, Dallas, TX, USA; ^3^Professor of Cardiology, Texas A&M University, College Station, TX, USA

**Background**: Borzoi dogs reportedly experience sudden death.

**Objectives**: To report electrocardiogram (ECG) intervals, amplitudes, and frequency of ECG abnormalities in clinically healthy Borzoi.

**Animals**: Eighty‐two client‐owned Borzoi.

**Methods**: Clinically healthy Borzoi were prospectively recruited as part of a larger cohort study and underwent an echocardiogram and an ECG. Borzoi with structural cardiac abnormalities were excluded. Standard ECG measurements were obtained. QT interval was corrected (QTc) using the Van de Water formula.

**Results**: Of 82 Borzoi with a structurally normal echocardiogram, ventricular arrhythmias were seen in six dogs and supraventricular premature complexes in one dog. Median P wave duration was 55 ms (range: 45‐70 ms). Median PR interval was 125 ms (range: 80‐175 ms), with 31 Borzoi (37.8%) having first degree atrioventricular block (PR interval >130 ms). Median QRS duration was 65 ms (range: 48‐90 ms). The median QT interval was 235 ms (range: 185‐275 ms). The median QTc interval was 270 ms (range: 223‐304 ms). Twenty‐nine of 82 Borzoi had a QT interval >240 ms (35.4%). Seventeen of 82 Borzoi (20.7%) had an abnormality of the ST segment, most commonly convexity/doming. Convexity of the ST segment was intermittent in nine dogs and persistent in four.

**Conclusions and Clinical Importance**: Prolonged QT intervals and ST segment abnormalities are not infrequent in clinically healthy Borzoi with normal echocardiograms. Whether these ventricular repolarization abnormalities relate to the reported predisposition for sudden death warrants further investigation. P, PR, and QRS durations are also commonly prolonged compared to reference intervals in other dogs.

## Oral rapamycin therapy in feline subclinical hypertrophic cardiomyopathy: Results of the RAPACAT clinical trial

### **Joshua A. Stern** ^1^; Joanna Kaplan^1^, DVM, DACVIM (Cardiology); Ashley Walker^1^, DVM; Victor Rivas^1^, BSc; Louise Grubb^2^, BSc, MBS; Aisling Farrell^2^, MPSI, MSc, PGCert; Stuart Fitzgerald^2^, MVB, MANZCVS; Carina Juaregui^1^, RVT, RLAT; Chris McLaughlin^3^ , DVM, DACVECC; Rachel Van Zile^3^, DVM; Teresa DeFrancesco^3^ , DVM, DACVECC, DACVIM (Cardiology); Kathryn Meurs^4^, DVM, PhD, DACVIM (Cardiology)

#### ^1^School of Veterinary Medicine, University of California‐Davis, Davis, CA, USA; ^2^TriviumVet, Waterford, Ireland; ^3^North Carolina State University, Raleigh, NC, USA; ^4^Dean, North Carolina State University, Raleigh, NC, USA

**Background**: Feline hypertrophic cardiomyopathy (HCM) remains a disease with little therapeutic advancement. Rapamycin modulates the mTOR pathway and prevents or reverses cardiac hypertrophy in rodent disease models. Its use in human renal allograft patients is associated with reduced cardiac wall thickness.

**Hypothesis/Objectives**: We sought to evaluate the effects of once‐weekly, delayed‐release (DR) rapamycin over 6 months on echocardiographic, biochemical, and biomarker responses in cats with subclinical, non‐obstructive HCM.

**Animals**: Client‐owned cats (n = 36) with subclinical HCM.

**Methods**: Cats enrolled in this double‐blind, multicenter, randomized, placebo‐controlled clinical trial were randomized to low‐ or high‐dose DR rapamycin or placebo. Cats underwent physical examination, quality of life assessment, blood pressure, hematology, biochemistry, total T4, fructosamine, urinalysis, N‐terminal pro‐brain natriuretic peptide, and cardiac troponin I at baseline, days 60, 120, and 180. Echocardiograms were performed at all time points excluding day 120. Outcome variables were compared using RMANCOVA.

**Results**: No demographic, echocardiographic, or clinicopathologic values were significantly different between study groups at baseline confirming successful randomization. At day 180, the primary study outcome variable, maximum LV myocardial wall thickness at any location, was significantly lower in the low‐dose DR rapamycin group compared to placebo (*P* = .01). Oral DR rapamycin was well tolerated in all cats with no significant differences in adverse events between groups.

**Conclusions and Clinical Importance**: Results demonstrate that DR rapamycin was well tolerated and may reverse or prevent progressive LV hypertrophy in cats with subclinical HCM. A future pivotal clinical trial is warranted.

## EQUINE

## *Staphylococcus aureus* nasal colonization and risk factors in horses admitted to a Veterinary Teaching Hospital

### **Marion Allano** ^1^; Julie‐Hélène Fairbrother^2^, DMV, MSc, DACVM; Marie Archambault^3^, DMV, MSc, PhD, DACVM; Julie Arsenault^4^, DMV, MSc, PhD; Frédéric Sauvé^5^, DMV, MSc, DACVD

#### ^1^Université de Montréal, Montreal, QC, Canada; ^2^Microbiologist, MAPAQ; ^3^Professor, Microbiology, Pathology and Microbiology, Université de Montréal, Montreal, QC, Canada; ^4^Professor, Epidemiology, Pathology and Microbiology, Université de Montréal, Montreal, QC, Canada; ^5^Professor, Dermatology, Clinical Sciences, Université de Montréal, Montreal, QC, Canada

**Background**: Methicillin‐resistant *Staphylococcus aureus* (MRSA) is a major cause of nosocomial infections, including in veterinary settings. Surveillance data are an important tool in the development of infection control policies.

**Hypothesis/Objectives**: This study aimed to investigate the prevalence and risk factors for *Staphylococcus aureus* (SA) and MRSA nasal colonization and the duration of the MRSA colonization.

**Animals**: Elective cases admitted to the veterinary teaching hospital were recruited (228 horses).

**Methods**: A cross‐sectional study was conducted over 3 years. Nasal swabs were collected at admission and cultured for SA. Methicillin‐resistant isolates were identified with MALDI‐TOF and confirmed with chromogenic agars and an oxacillin minimal inhibitory concentration testing. Horses colonized with MRSA were resampled until two negative cultures were obtained. Stabling management, activity, and medical history were obtained from owners via survey and medical files. Multivariate logistic regressions were used to model associations between risk factors and colonization.

**Results**: The prevalence of SA and MRSA nasal carriage was 18.4% (95% CI: 13.4%‐23.5%) and 5.7% (95% CI: 2.7%‐8.7%), respectively. Of the 13 horses colonized by MRSA, only one tested positive after 3 months of follow‐up. The risk factors associated with MRSA carriage were number of horses stabled with (more than 10 horses: OR 5.9; 95% CI: 1.1‐63.3), previous hospitalization (OR 6.1; 95% CI: 1.0‐35.7), and the year of admission (2022 vs. 2020‐2021; OR 8.8; 95% CI: 1.7‐90.9).

**Conclusions and Clinical Importance**: The prevalence of MRSA nasal colonization is of concern; however, the carriage is transient. Environmental risk factors are important.

## Effect of thyrotropin releasing hormone and pergolide on plasma β‐endorphin concentrations in horses and ponies

### **Nicholas Bamford** ^1^; Nicolas Galinelli^1^; Madison Erdody^1^; Skye Mackenzie^1^; Tobias Warnken^2^; Johanna Sonntag^2^; Patricia Harris^3^; Martin Sillence^4^; Simon Bailey^1^

#### ^1^The University of Melbourne, Parkville, VIC, Australia; ^2^Boehringer Ingelheim Vetmedica GmbH; ^3^Waltham Petcare Science Institute, Leicestershire, England, UK; ^4^Queensland University of Technology, Brisbane City, QLD, Australia

**Background**: β‐endorphin is a peptide hormone produced by the pituitary pars intermedia, and plasma concentrations may be increased with pituitary pars intermedia dysfunction (PPID). The potential value of measuring β‐endorphin for the diagnosis and/or monitoring of PPID has yet to be determined.

**Objectives**: To evaluate the response of plasma β‐endorphin concentrations to the thyrotropin releasing hormone (TRH) stimulation test and pergolide treatment, in horses and ponies with and without PPID.

**Animals**: Sixteen horses and ponies; eight with PPID and eight without PPID, matched by age and breed.

**Methods**: Animals underwent a standard TRH stimulation test (1 mg IV if ≥250 kg or 0.5 mg IV if <250 kg), before receiving pergolide (2 μg/kg PO SID) or no drug for 4 weeks in a randomized crossover design. Plasma β‐endorphin and ACTH concentrations were measured by radioimmunoassay and chemiluminescent immunoassay, respectively. Treatment effects were evaluated using Wilcoxon signed‐rank tests and Spearman rank correlation, with significance accepted at *P* < .05.

**Results**: Basal plasma β‐endorphin concentrations were significantly higher in PPID animals compared to non‐PPID animals (median [range], 1503 [572‐4907] pg/mL vs. 508 [207‐689] pg/mL; *P* = .003). Both groups demonstrated an increase in plasma β‐endorphin concentrations 10 minutes after TRH administration, which correlated with an increase in plasma ACTH concentrations (*r*s = 0.91; *P* < .001). Plasma β‐endorphin concentrations decreased following pergolide treatment in PPID animals (*P* = .008) but were unchanged in non‐PPID animals (*P* = .74).

**Conclusions and Clinical Importance**: Measurement of plasma β‐endorphin concentration may be a useful adjunct marker in the diagnosis and monitoring of PPID, and warrants further investigation.

## Effects of the sodium‐glucose cotransporter‐2 inhibitor velagliflozin on insulin concentrations in horses with insulin dysregulation

### **Nicholas Frank** ^1^; Kristen Thane^1^, DVM, DACVIM; Rebecca Voth^2^; Rebecca Klee^3^; Tobias Warnken^3^

#### ^1^Cummings School of Veterinary Medicine, Tufts University, North Grafton, MA, USA; ^2^Boehringer Ingelheim Animal Health USA, Inc.; ^3^Boehringer Ingelheim Vetmedica GmbH

**Background**: Sodium‐glucose cotransporter‐2 inhibitors (SGLT‐2i) warrant investigation as a therapeutic for insulin dysregulation.

**Hypothesis/Objectives**: Velagliflozin, a novel SGLT‐2i, decreases insulin concentrations in horses with moderate to severe insulin dysregulation (ID).

**Animals**: Privately owned adult equids (n = 37) with moderate to severe ID (oral sugar test insulin concentration >75 μIU/mL); 89% with a history of laminitis.

**Methods**: A double‐blinded placebo‐controlled trial was performed with horses randomly assigned to receive placebo (n = 19) or velagliflozin 0.3 mg/kg PO q24h (n = 18) for 20 weeks. During a second 20‐week period all horses received velagliflozin. Physical examinations and blood collections were performed at 0, 2, 4, 8, 12, 20, 22, 24, 28, 32, and 40 weeks. Placebo and velagliflozin groups were compared (Wk 0‐20). A mixed model for repeated measures ANOVA was used to assess effects of treatment, time, and treatment × time. Significance was set at *P* = .05.

**Results**: Day 0 insulin concentrations did not differ significantly between placebo and velagliflozin groups (*P* = .43). Treatment (*P* = .01), time (*P* < .01), and treatment × time (*P* < .01) effects were significant for plasma insulin concentrations when groups were compared Wk 0 to 20. Plasma insulin concentrations were significantly lower in the velagliflozin group, compared to the placebo group within 2 weeks (*P* = .03). All horses exhibited an increase in serum triglyceride concentrations during velagliflozin treatment, but development of hypertriglyceridemia was not accompanied by clinical abnormalities (lethargy, anorexia).

**Conclusions**: Velagliflozin significantly decreased basal plasma insulin concentrations in horses with moderate to severe ID in this trial.

## Xylazine and morphine caudal epidural analgesia in horses: Influence of volume on location of effect

### **Emma B. Gorenberg** ^1^; Dario Floriano^2^, DVM, DACVAA; Klaus Hopster^3^, DrMedVet, PhD, DECVAA

#### ^1^New Bolton Center, University of Pennsylvania, Philadelphia, PA, USA; ^2^Assistant Professor of Clinical Anesthesiology, School of Veterinary Medicine, University of Pennsylvania, Philadelphia, PA, USA; ^3^Associate Professor of Large Animal Anesthesiology, New Bolton Center, University of Pennsylvania, Philadelphia, PA, USA

**Background**: Caudal epidural analgesia is an important modality in the treatment of pain in the horse, though the influence of dilution volume on the location of effect requires further elucidation.

**Hypothesis/Objectives**: Objectives of the study were to describe the influence of dilution on the regional effects of xylazine and morphine epidural analgesia, with the hypothesis that increasing volumes produce different patterns and cranial spread of analgesia as determined by wireless thermal threshold (TT) testing.

**Animals**: Six university‐owned research/teaching horses (two mares, four geldings), aged 6 to 19 years and weighing 420 to 560 kg, that were deemed healthy on physical examination and basic lameness evaluation.

**Methods**: Prospective, randomized, blinded, cross‐over experimental study. Following routine placement of a caudal epidural catheter, all animals were subsequently instrumented with a TT *t*esting system at the left and right withers (location A) cranial (location B) and caudal (location C) abdominal area, over the tuber coxae (location D), and the hind limb dorsal pasterns (location E). All horses underwent five testing cycles with 0.2 mg/kg morphine and 0.2 mg/kg xylazine diluted to 20, 35, 50, 75, and 100 mL with a randomized order of treatment. TT testing was performed at 2, 4, 6, 8, and 10 hours by a blinded investigator.

**Results**: Timepoints were compared via paired *t* test to the baseline control (*P* < .05). Results are summarized in Table 1.

**Table:** TABLE 1 Mean and SD of thermal thresholds determined in six horses at the withers (location A), cranial (location B) and caudal abdominal area (location C), the area over the *tuber coxae* (location D) and the hind limb dorsal pastern regions (location E) after administration of 0.2 mg/kg morphine and 0.2 mg/kg xylazine diluted to different volumes.

Time (h)	Location A		Location B		Location C		Location D		Location E	
	Mean	SD	Mean	SD	Mean	SD	Mean	SD	Mean	SD
*25 mL volume administered*										
0	48.6	2.9	49.9	202	47.9	1.1	47.5	2.4	47.9	3.7
2	49.4	1.1	49.6	2.9	47.5	0.9	51.9	3.0	**55.0** *	**0.6**
4	50.7	2.9	47.9	3.7	49.8	3.2	48.6	2.9	**56.0** *	**0.0**
6	49.5	3.6	46.8	3.2	47.9	1.9	49.4	1.1	**54.6** *	**1.0**
8	49.6	4.8	47.9	1.9	48.7	2.1	50.7	2.9	**54.7** *	**0.8**
10	48.0	4.5	48.7	2.1	48.8	0.7	49.5	3.6	**53.9** *	**3.3**
*35 mL volume administered*										
0	48.2	2.8	49.9	2.2	47.9	1.1	47.7	2.4	47.9	3.7
2	48.2	3.1	48.6	1.1	47.5	0.9	**52.8** *	**2.8**	**55.2** *	**0.4**
4	50.0	2.7	47.0	2.7	46.8	3.2	**52.9** *	**2.9**	**55.2** *	**0.4**
6	49.2	4.0	47.0	3.2	47.9	1.9	**52.3** *	**2.3**	**54.9** *	**1.2**
8	50.4	4.7	49.0	2.9	48.7	2.1	**53.9** *	**1.9**	**54.8** *	**1.0**
10	48.5	4.6	48.0	2.2	48.0	0.7	51.2	4.5	**53.9** *	**3.3**
*50 mL volume administered*										
0	48.6	2.9	49.2	1.5	47.5	1.3	47.5	2.4	47.9	3.7
2	49.4	1.1	49.6	2.9	50.3	3.4	**53.5** *	**1.6**	55.3	0.5
4	50.7	2.9	47.9	3.7	**51.1** *	**5.3**	**52.3** *	**4.9**	**55.3** *	**0.5**
6	49.5	3.6	46.8	3.2	**51.6** *	**3.3**	**53.6** *	**3.4**	**54.9** *	**1.2**
8	49.6	4.8	47.9	1.9	**52.7** *	**2.6**	**53.2** *	**3.1**	**55.0** *	**1.1**
10	48.0	4.5	48.7	2.1	50.8	2.9	**54.5** *	**2.9**	**54.9** *	**2.6**
*75 mL volume administered*										
0	47.6	2.4	50.2	1.3	48.3	1.5	47.5	2.4	47.9	3.7
2	50.4	1.6	**53.3** *	**3.0**	**54.0** *	**4.0**	**55.5** *	**0.5**	**55.3** *	**0.5**
4	50.1	2.9	51.5	5.6	**54.7** *	**2.3**	**54.5** *	**2.2**	**55.2** *	**0.4**
6	48.9	3.1	50.4	6.0	**54.5** *	**2.7**	**54.3** *	**2.2**	**54.9** *	**1.2**
8	46.8	2.5	51.3	4.5	**54.3** *	**3.1**	**53.9** *	**2.7**	**55.0** *	**2.6**
10	47.4	4.1	**52.5** *	**3.7**	**54.3** *	**3.1**	**53.4** *	**4.5**	**54.9** *	**2.6**
*100 mL volume administered*										
0	48.1	3.0	48.5	1.4	47.9	1.1	47.5	2.4	49.0	2.3
2	50.8	2.5	**52.9** *	**3.0**	**53.8** *	**4.1**	**55.5** *	**0.5**	**55.3** *	**0.5**
4	**51.0** *	**3.3**	**52.1** *	**3.3**	**54.5** *	**2.3**	**53.2** *	**5.0**	**55.3** *	**0.5**
6	51.0	4.0	**51.9** *	**3.9**	**54.2** *	**2.8**	**54.8** *	**22**	**55.3** *	**0.5**
8	**51.3** *	**3.4**	51.5	4.3	**54.2** *	**3.3**	**54.7** *	**2.4**	**55.3** *	**0.5**
10	**52.2** *	**2.1**	51.7	4.1	**54.3** *	**3.2**	**54.7** *	**0.5**	**56.0** *	**0.0**

* Statistically significant different (*P* < .05) to the baseline measurements (time 0 hours).

**Conclusions and Clinical Importance**: Increasing volume influences the regional effects of caudal epidural analgesia in horses, though efficacy and reliability of analgesia may be reduced when treating more cranial loci of pain.

## Insulin and metformin modulate equine neutrophil ROS production ex vivo

### **Shune Kimura** ^1^; Roya Shirzad^1^; Londa Berghaus^1^; Kelsey Hart^2^, DVM, PhD, DACVIM (LAIM)

#### ^1^University of Georgia, Athens, GA, USA; ^2^Large Animal Medicine, University of Georgia, Athens, GA, USA

**Background**: Systemic inflammatory response syndrome (SIRS) is associated with many equine diseases. Therapeutic strategies for equine SIRS are limited and an urgent research need. Insulin dysregulation is observed in SIRS and contributes to case morbidity and mortality. Oxidative damage and reactive oxygen species (ROS) produced by neutrophils influence immune and insulin dysregulation in SIRS. The insulin‐sensitizing drug metformin has demonstrated beneficial metabolic and immunomodulatory effects in rodent SIRS/sepsis models and human patients with concurrent insulin dysregulation and sepsis. Currently, metformin is only used in chronic insulin resistance for horses, and effects on equine leukocyte function are unknown.

**Hypothesis/Objectives**: Insulin and metformin treatment will modulate ROS production in activated equine neutrophils.

**Animals**: Six healthy adult horses.

**Methods**: Neutrophils were isolated from peripheral blood via density‐gradient centrifugation. Ex vivo ROS production was stimulated with phorbol myristate acetate, killed whole‐cell *Staphylococcus aureus*, and lipopolysaccharide in the presence and absence of insulin (25, 50, 100, and 200 μIU/mL) and metformin (0.075, 0.75, and 7.5 mM) alone and in combination. A previously validated fluorometric assay was used to measure ROS production.

**Results**: Insulin and metformin both significantly reduced stimulant‐induced ROS production by 26% to 42% (*P* = .001‐.04) and 3% to 20% (*P* = .001‐.02) respectively. In combination, metformin enhanced insulin's reduction of neutrophil ROS production by 5.5% to 33% following activation (*P* range: .001‐.02).

**Conclusions and Clinical Importance**: These data support our hypothesis that insulin and insulin‐sensitizing drug metformin have direct immunomodulatory effects on equine neutrophils. Further ex vivo and in vivo studies should investigate whether metformin modulates other aspects of equine immune function.

## Bile acid profiling of pyloric gastric fluid in horses with equine glandular gastric disease

### **Linda J. Paul** ^1^; Heidi Banse^1^; Frank Andrews^1^; Aaron Ericsson^2^

#### ^1^Louisiana State University, Baton Rouge, LA, USA; ^2^University of Missouri, Columbia, MO, USA

**Background**: The etiology of hyperemic equine glandular gastric disease (EGGD) remains poorly characterized. In people, reflux of bile into the stomach can cause bile gastropathy and has many parallels to hyperemic EGGD.

**Hypothesis/Objectives**: We hypothesize that there is an increase in bile acid concentrations with an increased proportion of conjugated bile acids in the pyloric gastric fluid associated with hyperemic EGGD. Our specific objective is to compare the bile acid profiles of pyloric gastric fluid between horses with and without hyperemic EGGD.

**Animals**: Horses from the institutional herd underwent gastroscopic evaluation. Enrolled horses were designated as control (no abnormalities observed, n = 8) or affected (hyperemic EGGD observed, n = 15). Of the 15 horses with hyperemic EGGD, lesions (disruption of the mucosal surface) were also observed in seven.

**Methods**: This was a case–control study in horses with naturally occurring EGGD. During gastroscopic evaluation, gastric fluid was collected from the pylorus. Bile acid concentrations were measured using custom ultra‐performance liquid chromatography coupled with mass spectrometry (UPLC/MS). Concentrations were compared between groups using a *t*‐test or Mann‐Whitney *U* test.

**Results**: Thirteen bile acids were analyzed: 10 had measurable concentrations in all horses (n = 23), 2 in 22 horses, and one in only 3 horses. Affected horses had increased concentrations of taurolithocholic acid (*P* = .04), lithocholic acid (*P* = .02), and hyodeoxycholic acid (*P* = .002) compared to controls. There was no difference detected in the percentage of conjugated or unconjugated bile acids between groups.

**Conclusions and Clinical Importance**: These results support a role of bile acid reflux in pyloric, hyperemic EGGD.

## Successful establishment of equine duodenal organoids from endoscopically‐guided biopsies

### **Breanna Sheahan** ^1^; Lara Madding^2^, PhD

#### ^1^College of Veterinary Medicine, North Carolina State University, Raleigh, NC, USA; ^2^Research Coordinator, North Carolina State University, Raleigh, NC, USA

**Background**: Intestinal organoids are utilized for understanding epithelial biology, disease mechanisms, and drug metabolism in human medicine. They are increasingly important for personalized and precision medicine. To date, all published methods of producing equine intestinal organoids use tissue obtained post‐mortem or via exploratory celiotomy, which limits the utility of this model.

**Hypothesis**: Duodenal organoids will be cultured from duodenal endoscopically‐guided biopsies performed in standing sedating horses.

**Animals**: Five healthy adult mares (age range: 12‐21 years, 4 TB and 1 QH) from NC State Research herds.

**Methods**: After fasting, horses were sedated and gastroscopy was performed with a 3.25 m endoscope. After passing into the proximal duodenum, 6‐8 biopsies were obtained using 2.3 mm serrated biopsy forceps (Figure 1A,B). Duodenal crypts were separated from the underlying lamina propria by mechanical and chemical dissociation (Figure 1D‐F). Crypts were then filtered and plated in a 3D matrix (Matrigel). After polymerization, media was applied to the cultures and they were incubated at 37°C in 5% CO_2_. Organoid growth was monitored daily and media was changed as necessary. Media modifications with varying concentrations of R‐spondin, a Wnt signaling molecule, were performed to test the requirements for organoid growth.

**Figure:**
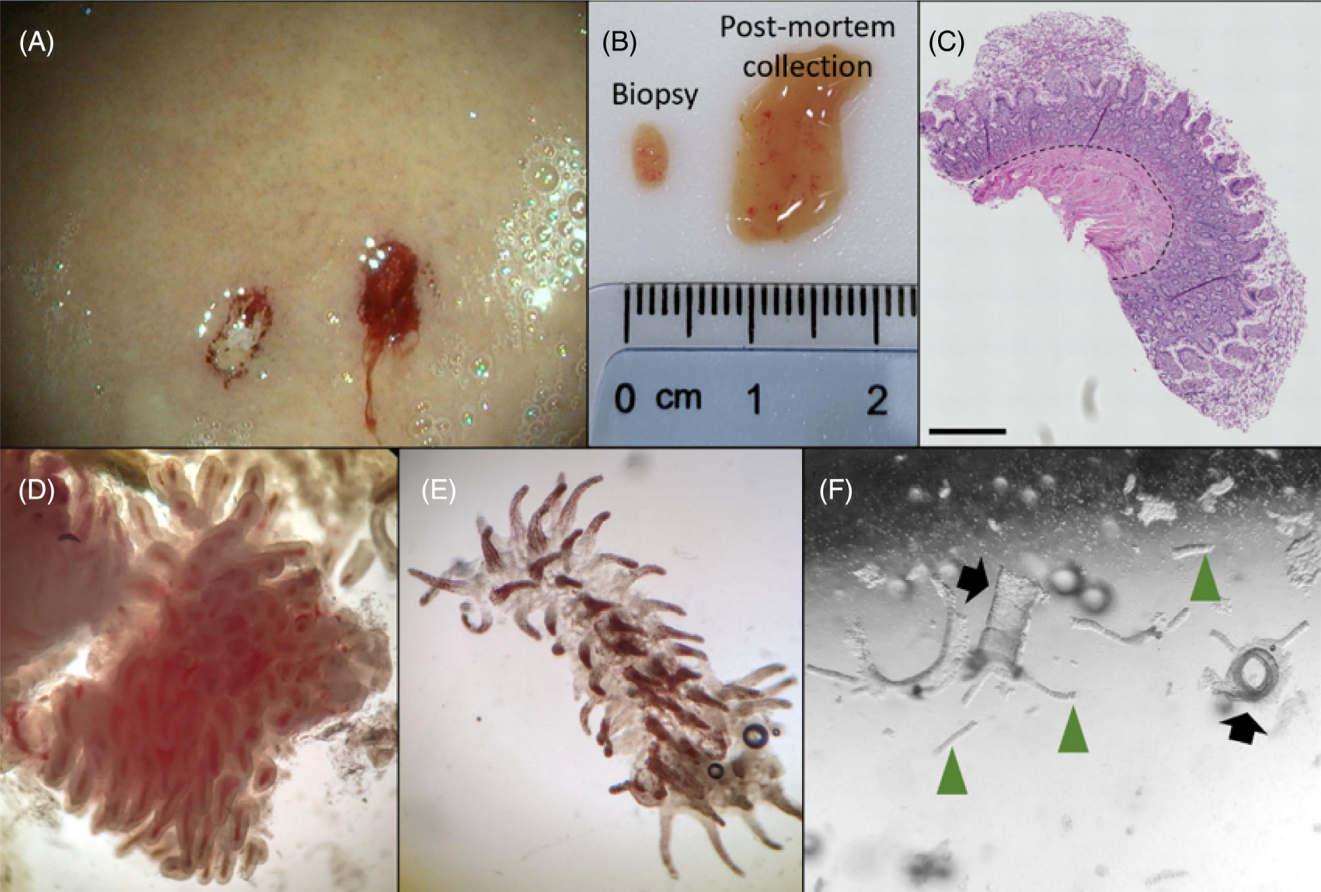
FIGURE 1. Acquisition of duodenal crypts from endoscopically‐guided biopsies. (A) Video endoscope image post‐biopsy acquisition (2) in duodenum of a health horse. (B) Tissue size comparison of biopsy sample vs typical tissue sample used for crypt isolation, collected postmortem. (C) Hematoxylin and Eosin‐stained cross section of duodenal biopsy demonstrating adequate tissue depth. Base of crypt epithelium indicated by dashed black line. Scale bar 500 μm. (D,E) stereomicroscopy images of duodenal biopsy pre‐isolation, lamina propria post‐isolation, and epithelium post‐isolation, respectively (crypts, green arrowheads; villi, black arrows).

**Results**: Duodenal organoids were successfully cultured from endoscopically‐guided biopsies from adult horses (Figure 1). Sufficient crypts for culture were obtained in 4/5 horses. R‐spondin supplementation is required for duodenal organoid growth (Figure 2).

**Figure:**
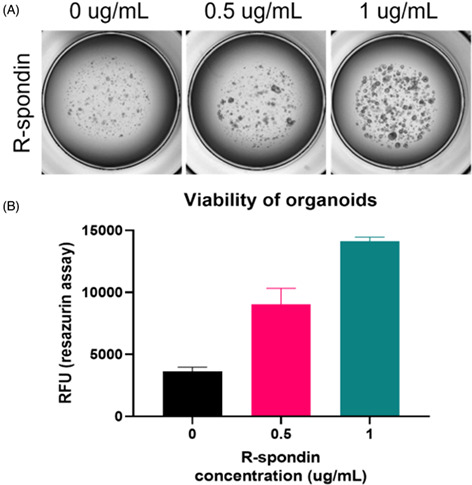
FIGURE 2. Optimization of media conditions for equine duodenal organoids. (A) representative images of organoid cultures after 5 days of treatment with the indicated concentrations of R‐spondin, a Wnt signaling molecule. Magnification of 2×. (B) Relative fluorescence units (RFU) of resazurin reduction, an indicator of viable cells. Resazurin assay performed 5 days after initial supplementation with indicated concentrations of R‐spondin (mean ± SD, n = 3).

**Conclusions and Clinical Importance**: This study demonstrates duodenal organoid establishment from live adult horses, allowing for personalized medicine approaches based on organoid assays.

## FOOD ANIMAL

## Pharmacokinetics and efficacy of pantoprazole in sheep after intravenous and subcutaneous administration

### **Joe S. Smith** ^1^; Kailee Bennet^2^; Pierre‐Yves Mulon^3^, DVM, DES, DACVS; Jessica Gebert^4^, MS; Joan Bergman^5^, MS; Jessica Garcia^2^, DVM; Olivia Escher^2^; Lainey Harvill^5^; Ryan Flynn^2^; Sherry Cox^6^, MS, PhD

#### ^1^University of Tennessee, Knoxville, TN, USA; ^2^Large Animal Clinical Sciences, University of Tennessee, Knoxville, TN, USA; ^3^Associate Professor, Large Animal Clinical Sciences, University of Tennessee, Knoxville, TN, USA; ^4^College of Veterinary Medicine, Lincoln Memorial University, Harrogate, TN, USA; ^5^Biomedical and Diagnostic Sciences, University of Tennessee, Knoxville, TN, USA; ^6^Professor, Biomedical and Diagnostic Sciences, University of Tennessee, Knoxville, TN, USA

**Background**: Sheep suffer from abomasal ulcers caused by factors like diet, nonsteroidal anti‐inflammatory drug (NSAID) treatment, and stress with limited therapies. Pantoprazole is a proton pump inhibitor (PPI) used for gastric ulcers, gastritis, and esophagitis in humans, and can be administered by injection.

**Objectives**: The objectives of this study were to determine the pharmacokinetics of pantoprazole after intravenous (IV) and subcutaneous (SC) administration, as well as to determine the effect of abomasal pH after administration.

**Animals**: Four adult ewes, with abomasal cannulations.

**Methods**: Ewes were administered a 1 mg/kg IV or SC dose of pantoprazole once daily for three consecutive days. Blood samples were collected from the ewes over 24 hours after the first administration, and abomasal fluid samples were collected over 96 hours. Plasma concentrations were analyzed via reversed‐phase liquid chromatography. Data was interpreted with commercial software.

**Results**: Pantoprazole was eliminated rapidly, with a 3.3‐hour IV elimination half‐life after IV. Both routes of administration had similar values for Area under the Curve: 17 766 h ng/mL (IV), 14 961 h ng/mL (SC) with a SC bioavailability of 88%. The abomasal fluid pH was significantly (*P* < .05) higher than pre‐pantoprazole pH levels up to 8 hours after dosing on all 3 days for both treatments.

**Conclusions**: Pantoprazole offers a potential therapy for gastroprotection in sheep. Similarities in IV and SC administration parameters suggest that SC administration may be efficacious enough to allow for easier dosing for practitioners and clients.

## The pharmacokinetics and efficacy of intravenous single dose esomeprazole in sheep

### **Joe S. Smith** ^1^; Jessica Gebert^2^, MS; Kailee Bennet^3^; Lisa Ebner^4^, DVM, MS, DACVAA; Ryan Flynn^5^; Pierre‐Yves Mulon^6^, DVM, DES, DACVS; Lainey Harvill^7^; Olivia Escher^5^; Amanda Kreuder^8^; Joan Bergman^7^, MS; Sherry Cox^7^, MS, PhD

#### ^1^University of Tennessee, Knoxville, TN, USA; ^2^College of Veterinary Medicine, Lincoln Memorial University, Harrogate, TN, USA; ^3^Lieutenant, Large Animal Clinical Sciences, University of Tennessee, Knoxville, TN, USA; ^4^Associate Professor, College of Veterinary Medicine, Lincoln Memorial University, Harrogate, TN, USA; ^5^Large Animal Clinical Sciences, University of Tennessee, Knoxville, TN, USA; ^6^Associate Professor, Large Animal Clinical Sciences, University of Tennessee, Knoxville, TN, USA; ^7^Biomedical and Diagnostic Sciences, University of Tennessee, Knoxville, TN, USA; ^8^Veterinary Microbiology and Preventive Medicine, Iowa State University, Ames, IA, USA

**Background**: Abomasal ulceration presents a morbidity in sheep, and currently there is a lack of pharmacokinetic and pharmacodynamic data for proton pump inhibitors reported for use in sheep. The proton pump inhibitor esomeprazole has been used in small animal and human patients for gastroprotection via decreasing acid secretion.

**Objective**: To report pharmacokinetic parameters and pharmacodynamic effect of esomeprazole in sheep after single dosing (1.0 mg/kg, intravenous).

**Animals**: Four healthy adult ewes.

**Methods**: Blood samples were collected at 15 time points over a 24‐hour period after intravenous dosing. Samples were analyzed for concentrations of esomeprazole and the metabolite esomeprazole sulfone by high performance liquid chromatography. Abomasal fluid was sampled 10 times over 24 hours before and after drug administration. Pharmacokinetic and pharmacodynamic data were evaluated with commercial software.

**Results**: Esomeprazole was rapidly eliminated. Elimination half‐life, area under the curve, initial concentration and clearance were 0.2 hour, 1197 h ng/mL, 4321 ng/mL, and 0.83 mL/h/kg, respectively. For the sulfone metabolite elimination half‐life, area under the curve and maximum concentration were 0.16 hour, 22.5 h ng/mL, and 65.0 ng/mL, respectively. Abomasal pH was significantly elevated from 1 to 6 hour after administration and remained above 4 for at least 8 hours after administration. No adverse effects were noted in the study ewes.

**Conclusions**: Esomeprazole was rapidly eliminated in sheep, similar to goats. Abomasal pH was increased, but future studies will be necessary to develop a clinical management approach to the use of esomeprazole in sheep.

## Tulathromycin metaphylaxis increases isolation of multidrug resistant *Mannheimia haemolytica* in stocker heifers

### **Amelia R. Woolums** ^1^; William Crosby^2^, DVM; Brandi Karisch^3^, PhD; Lari Hiott^4^; Alexandra Pittman^5^; Jonathan Frye^4^, PhD; Charlene Jackson^4^, PhD; Lee Pinnell^6^, PhD; William Epperson^7^, DVM, MS, DACVPM (Epi); John Blanton^8^, PhD; Sarah Capik^9^, DVM, PhD, DACVPM; Paul Morley^10^, DVM, PhD, DACVIM (LAIM)

#### ^1^College of Veterinary Medicine, Mississippi State University, Starkville, MS, USA; ^2^Graduate Research Assistant, Clinical Instructor, Pathobiology and Population Medicine, College of Veterinary Medicine, Mississippi State University, Starkville, MS, USA; ^3^Associate Professor, Animal and Dairy Science, Mississippi State University, Starkville, MS, USA; ^4^Research Microbiologist, Bacterial Epidemiology and Antimicrobial Resistance Research, Agricultural Research Service, United States Department of Agriculture; ^5^Instructor, Animal and Dairy Science, Mississippi State University, Starkville, MS, USA; ^6^Postdoctoral Research Associate, Veterinary Education, Research and Outreach, Texas A&M University, College Station, TX, USA; ^7^Professor and Department Head, Pathobiology and Population Medicine, College of Veterinary Medicine, Mississippi State University, Starkville, MS, USA; ^8^Professor and Department Head, Animal Sciences, Purdue University, West Lafayette, IN, USA; ^9^Tumbleweed Veterinary Services, PLLC; ^10^Professor and Director of Research, Veterinary Education, Research, and Outreach, Texas A&M University, College Station, TX, USA

**Background**: Bovine respiratory disease (BRD) is commonly controlled by metaphylaxis; increasing prevalence of antimicrobial resistant (AMR) *Mannheimia haemolytica* (MH) may decrease efficacy.

**Hypothesis/Objectives**: Determine effect of tulathromycin metaphylaxis on: (a) AMR of MH isolated, and (b) health of high‐risk stocker calves at 3 (WK3) and 10 (WK10) weeks.

**Animals**: Auction‐derived beef cross heifers (n = 335, 232 ± 17.8 kg) purchased for 4 trials from 2019 to 2021.

**Methods**: Cattle were randomized and administered tulathromycin at 2.5 mg/kg SC (META, n = 168) or not (NO‐META, n = 167). Nasopharyngeal swabs were obtained at arrival, WK3, and WK10 for culture, susceptibility testing, whole genome sequencing (WGS), and AMR gene identification. Isolates were multidrug resistant (MDR) if they were not susceptible to \geq\ 3 antimicrobial classes.

**Results**: BRD morbidity was significantly lower in META (14.9%) animals than NO‐META (29.3%) (*P* = .002) but there was no difference in WK3 or WK 10 MH isolation between groups. Odds of MDR MH recovery was significantly higher in META at WK3 and WK10 (WK3: OR = 12.62, 95% CI: 5.42‐29.40, *P* < .0001; WK10: OR = 5.95, 95% CI: 1.35‐26.27, *P* = .02) compared to NO‐META, and treated animals at WK3 (OR = 6.00, 95% CI: 2.36‐15.26, *P* = .0002) compared to healthy. AMR genes were identified in more META (100%, n = 75) compared to NO‐META (52%, n = 31/60) isolates in WK3. Metagenomic assessment of swabs is underway.

**Conclusions**: Tulathromycin metaphylaxis was associated with increased MDR MH isolation in high‐risk heifers at WK3 and WK10. Isolation of MH was not decreased in META cattle. Metaphylaxis reduced morbidity, possibly through non‐antimicrobial mechanisms; work investigating such mechanisms could guide approaches that decrease BRD without increasing AMR.

## NEUROLOGY

## Surgical experience with lumbosacral (myelo)meningoceles with tethered cord syndrome (TCS) in 8 bulldog puppies

### **Patrick Roynard**

#### College of Veterinary Medicine, The Ohio State University, Columbus, OH, USA

**Background**: Limited information is reported regarding surgical management of lumbosacral (myelo)meningoceles in puppies, the largest series being six dogs from several surgeons.

**Hypothesis/Objectives**: Surgical management may be associated with neurological improvement. The author reports MRI features (entire neuraxis), personal surgical experience/findings (through VITOM® intra‐operative images/videos) and outcome.

**Animals**: Eight bulldog puppies diagnosed with (myelo)meningocele on MRI.

**Methods**: All the dogs examined by the author and diagnosed with (myelo)meningocele were offered surgical management. All dogs operated on are included in this cohort. The surgical intervention and findings will be reviewed through step‐by‐step intra‐operative images/videos.

**Results**: All dogs showed urinary/fecal incontinence, pelvic limbs deficits (most commonly sciatic nerve), and imaging ± clinical features of TCS. One dog (lipomyelomeningocele) died of a pulmonary fatty embolism within 72 hours of surgery, preventing follow‐up (necropsy findings presented). Adhesions between the dural sac and laminae of adjacent vertebrae, or within the meningocele between nervous structures and meninges, are frequent and should be addressed surgically. Three dogs recovered urinary/fecal continence with improvement of pelvic limbs deficits; four showed only mild/no improvement of urinary/fecal incontinence (three with moderate improvement of pelvic limbs deficits, one unchanged). One dog is only 2 months post‐operative at this time, all others >1 year follow‐up.

**Conclusions and Clinical Importance**: Surgery for congenital lumbosacral (myelo)meningoceles can be associated with neurological improvement. Review of the cases of this series and cases reported in the literature did not allow identification of prognostic factors (imaging, clinical or surgical) for urinary/fecal continence.

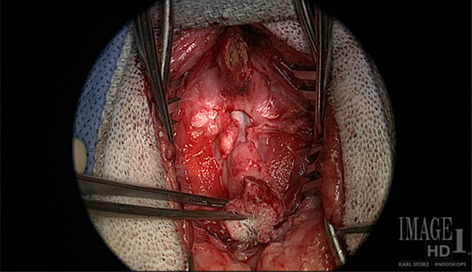

## Circulating microRNA profiles in dogs with acute spinal cord injury

### **Jihey Lim** ^1^; Sehwon Koh^2^, PhD; Alexa Stephen^3^, DVM; Joan Coates^4^, DVM, MS, DACVIM (Neurology); Sissy Hong^5^, DVM, MVM, MS, DACVIM (Neurology)

#### ^1^University of Missouri, Columbia, MO, USA; ^2^Assistant Professor, Veterinary Medicine and Surgery, University of Missouri, Columbia, MO, USA; ^3^Resident, Neurology/Neurosurgery, Veterinary Medicine and Surgery, University of Missouri, Columbia, MO, USA; ^4^Professor, Veterinary Medicine and Surgery, University of Missouri, Columbia, MO, USA; ^5^Assistant Teaching Professor, Veterinary Medicine and Surgery, University of Missouri, Columbia, MO, USA

Circulating microRNAs (miRNA) have emerged as useful biomarkers for their stability and tissue specificity. However, miRNA studies in dogs with acute spinal cord injury (SCI) from intervertebral disc herniation are limited.

We hypothesized that circulating miRNA reflects injury severity and cell type specificity in a severity‐dependent manner.

Twenty serum samples of 13 dogs with SCI with various severity from a hospital population and seven healthy control dogs.

Retrospective study. The miRNA was isolated and used for library preparation. Libraries were pooled and sequenced using NovaSeq SP‐PE50 to generate 10 million sequences. Post‐sequence pipeline was generated. Sample‐to‐sample distance map and principal component analysis (PCA) from each severity group were performed. Volcano plot analyses were performed to identify differentially expressed (DE) miRNAs. In silico analysis further categorized DE miRNAs by their potential cell type sources by cross‐referencing with miRNA database obtained from rodent motor neurons (MN), astrocytes (ASC), and microglia (MG).

PCA demonstrated severity‐dependent grouping of miRNA profiles. We identified 94 (mild), 134 (moderate), and 141 (severe) DE miRNAs including 48 (severe), 32 (moderate), and 15 (mild) unique to each severity. In the severe group, we identified 35 down‐regulated and 13 up‐regulated unique miRNAs that originated from ASC (32 miRNAs), MG (7 miRNAs), and MN (12 miRNAs).

Combined results indicate that circulating miRNAs are promising biomarkers to evaluate SCI injury severities. Further studies elucidating the role of cell type‐specific miRNA biomarkers and their correlation with the long‐term functional outcome will provide valuable knowledge to develop novel therapeutic approaches in the SCI.

## NUTRITION

## Starch:Protein ratio in energy expenditure and body composition in neutered and intact male cats

### **Aulus C. Carciofi** ^1^; Camila Goloni^2^; Leticia Pacheco^1^; Leticia Luis^1^; Stephanie Theodoro^1^; Lucas Scarpim^1^; Celina Torres^1^

#### ^1^São Paulo State University; ^2^Post doctoral, São Paulo State University

**Background**: Protein or starch intake may influence energy expenditure (EE) and body composition (BC) of male cats, this effect might differ between intact and neutered animals.

**Hypothesis/Objectives**: To compare the ad libitum intake of two kibble diets with different starch to protein ratios (but similar in fat and fiber) on body weight (BW), BC, EE, and physical activity (PA) of privately owned cats.

**Animals**: Non‐obese cats, intact male (IM) 1.6 ± 0.8 years (n = 10) and neutered male (NM) 2.2 ± 1.2 years (n = 9).

**Methods**: In a crossover design, cats were fed ad libitum for 4 months a high starch (HS; starch 40%, protein 38%) or high protein (HP: starch 20%, protein 55%) kibble diet. The BC and EE were evaluated by doubly labeled water method, and PA by 3‐axyal accelerometer. Results were submitted to ANOVA in a 2 diets × 2 sexual condition arrangement (*P* ≤ .05).

**Results**: The IM cats presented higher EE (481 ± 29 kJ/kg 0.67/day), lean mass (LM; 90% ± 0.8%) and PA than NM (EE: 382 ± 28 kJ/kg 0.67/day; LM: 84% ± 1.1%; *P* < .05). NM fed HS diet did not change BW, but their fatty mass reduced 21% (*P* = .04) and LM increased 4% (*P* < .01). The NM fed HP diet increased BW (5%) and LM (7%) (*P* < .05). No diet effect was observed for IM cats (*P* > .05). The EE increased 10% when cats were fed HP compared to HS diet (*P* < .05). The EE was positively associated with PA (Figure 1).

**Figure:**
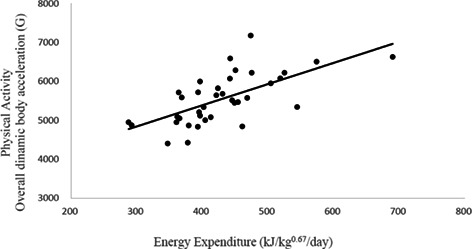
FIGURE 1. Energy expenditure vs physical activity, overall dynamic body acceleration (ODBA; G gravitational): *y* = 5.4443*x* + 3205; *R* ^2^ .42; *P* < .01; n = 37.

**Conclusions**: IM had higher LM, EE, and PA than NM non‐obese cats. In NM, the HS diet maintained BW and improved BC.

## Ionized hypercalcemia resolves with nutritional modification in cats with idiopathic hypercalcemia and chronic kidney disease

### **Valerie J. Parker**; Margot Ehrlich; Adam Rudinsky; Dennis Chew

#### The Ohio State University, Columbus, OH, USA

**Background**: Idiopathic hypercalcemia (IHC) and chronic kidney disease (CKD) are two common causes of ionized hypercalcemia in cats. Various nutritional management strategies to address hypercalcemia have been described with little consensus about best practice.

**Hypothesis**: Feeding a diet with <200 mg calcium per 100 kcal (kcal) and a dietary calcium:phosphorus (Ca:P) ratio <1.4:1 can effectively manage hypercalcemia in cats.

**Animals**: Cats with persistent ionized hypercalcemia diagnosed with either IHC (n = 7) or CKD (n = 2) were included.

**Methods**: Retrospective case series of cats with ionized hypercalcemia. Cats with neoplasia, primary hyperparathyroidism, or those receiving medications known to affect calcium concentrations (eg, alendronate, corticosteroids, furosemide) were excluded. Cats were included if nutritional recommendations to address the hypercalcemia were provided and if follow‐up ionized calcium (iCa) concentrations were obtained.

**Results**: Baseline iCa median (range) was 1.56 mmol/L (1.50‐1.65 mmol/L). At initial evaluation, 5 cats were fed a diet with >200 mg calcium/100 kcal, 3 cats were fed a diet with a Ca:P ratio >1.4:1, and 1 cat was fed a diet with both >200 mg calcium/100 kcal and a Ca:P ratio >1.4:1 (Table 1). Median (range) time from baseline to first recheck of iCa was 10.6 weeks (range, 3.0‐20.1 weeks). Ionized hypercalcemia completely resolved in 7/9 cats (78%) and improved in the remaining 2/9 cats (22%) (Figure 1).

**Table:** TABLE 1. Dietary calcium [mg per 100 kcal (kcal)] and calcium:phosphorus (Ca:P) ratios of diets fed to hypercalcemic cats at baseline and after modified dietary recommendations were made.

	Baseline diet characteristics		Modified dietary recommendation	
	Dietary calcium (mg/100 kcal)	Dietary Ca:P ratio	Dietary calcium (mg/100 kcal)	Dietary Ca:P ratio
Cat 1 (IHC)	441	1.2	161	1.2
Cat 2 (CKD)	160	1.5	140	1.1
Cat 3 (IHC)	262	1.3	173	1.1
Cat 4 (IHC)	160	1.6	170	1.0
Cat 5 (IHC)	317	1.3	188	1.2
Cat 6 (IHC)	130	1.6	158	1.0
Cat 7 (IHC)	213	1.0	140	1.1
Cat 8 (IHC)	217	1.4	173	1.1
Cat 9 (CKD)	215	1.7	120	1.2

*Note*: Cats were all fed diets that had a diet with <200 mg/100 kcal and a Ca:P ratio <1.4.

**Figure:**
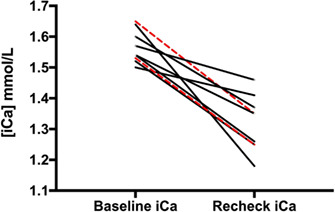
FIGURE 1. Ionized calcium (iCa) concentrations in hypercalcemic cats (black lines: IHC; red lines: CKD) at baseline and recheck evaluation following diet modification.

**Conclusions and Clinical Importance**: Cats with ionized hypercalcemia can return to normocalcemia when fed diets with <200 mg calcium/100 kcal as well as a dietary Ca:P ratio <1.4:1.

## ONCOLOGY

## The integrated canine data commons (ICDC): Two year experience report

### **Amy** **LeBlanc** ^1^ , DVM, DACVIM (Oncology); Erika Berger^2^; Gina Kuffel^3^; Philip Musk^4^; Deborah Knapp^5^, DVM, MS, DACVIM (Oncology); Dolores L. McCall^6^ ; Ralph Parchment^7^, PhD; Connie Sommers^8^, PhD; Toby Hecht^9^, PhD

#### ^1^Director, Comparative Oncology Program, Molecular Imaging Branch, National Cancer Institute; ^2^Frederick National Laboratory for Cancer Research, Frederick, MS, USA; ^3^Technical Project Manager, CTOS Program Management Office, Frederick National Laboratory for Cancer Research, Frederick, MS, USA; ^4^Business Analyst, Frederick National Laboratory for Cancer Research, Frederick, MS, USA; ^5^Distinguished Professor of Comparative Oncology; ^6^Professor of Comparative Oncology, Director‐Purdue Comparative Oncology Program, Co‐Section Head‐Oncology, Veterinary Clinical Sciences, Purdue University, West Lafayette, IN, USA; ^7^Senior Managing Director, Applied/Developmental Research Directorate, Frederick National Laboratory for Cancer Research, Frederick, MS, USA; ^8^Program Director, ImmunoOncology Branch, Developmental Therapeutics Program, National Cancer Institute; ^9^Deputy Director, Division of Cancer Treatment and Diagnosis, National Cancer Institute

**Background**: Highly relevant pre‐clinical animal models are essential to inform the most promising novel immuno‐oncology drug candidates to advance into human clinical trials. Often, canine cancers closely mimic their human counterparts. The NCI's Division of Cancer Treatment and Diagnosis launched the ICDC in 2020 to make data from spontaneous canine cancer accessible for hypothesis‐driven comparative analysis.

**Hypothesis/Objectives**: This report is an update after 2 years of ICDC development, informing the community of the new resources, features, and tools.

**Animals**: The ICDC contains the data for 598 dogs across 282 breeds, including 114 mixed breed dogs.

**Methods**: The ICDC is a publicly available, cloud‐based repository built upon a graph database. The flexible ICDC data model can add nodes and properties as needed. Intuitive features developed to support the findability of data, data submission, and downstream data analysis help further research on human cancers by enabling comparative analysis.

**Results**: The ICDC now contains over 33 TB of data for 857 unique samples. Key data types include pathology, WES, WGS, and RNA‐Seq from 551 malignant tumors across more than 14 histologies and 168 normal tissue samples. The ICDC user interface is under continuous development to improve user experience and assist researchers in building cohorts pertinent to their scientific questions.

**Conclusions/Clinical Importance**: Dogs offer a unique comparative model that can be used to interrogate response similarities to cancer therapies in dogs versus humans, including tumor microenvironment composition, the prevalence of cancer specific mutations, and the landscape of tumor profiles.

## Frequency of cancer following screening in higher risk, middle‐aged to older dogs

### **Jenna H. Burton** ^1^; Douglas Thamm^2^, DVM, DACVIM (Oncology); David Vail^3^, DVM, MS, DACVIM (Oncology); Jennifer Willcox^4^; Sami Al‐Nadaf^4^ ; Sarah Adrianowycz^5^; Rubi Hayim^6^; Kara Magee^5^; Rachel McMahon^5^ ; Ann Marie Picone^5^; Rachel Uyehara^5^; Stephen Johnston^7^

#### ^1^Flint Animal Cancer Center, Colorado State University, Fort Collins, CO, USA; ^2^Professor of Oncology, Flint Animal Cancer Center, Colorado State University, Fort Collins, CO, USA; ^3^Professor, Department of Medical Sciences, School of Veterinary Medicine, University of Wisconsin‐Madison, Madison, WI, USA; ^4^School of Veterinary Medicine, University of California‐Davis, Davis, CA, USA; ^5^Clinical Trials Intern, School of Veterinary Medicine, University of Wisconsin‐Madison, Madison, WI, USA; ^6^School of Veterinary Medicine, University of Wisconsin‐Madison, Madison, WI, USA; ^7^School of Life Sciences, Arizona State University, Tempe, AZ, USA

**Background**: Cancer screening is used to identify malignant tumors prior to development of clinical signs, with the aim that early cancer detection may lead to improved outcomes. To understand the potential benefits of cancer screening in dogs, a greater understanding of the prevalence of cancer in higher‐risk populations of dogs as well as which cancers are frequently identified are needed.

**Hypothesis/Objectives**: Determine the prevalence of undiagnosed malignant neoplasms in a cohort of healthy middle‐aged to older dogs.

**Animals**: A total of 913 healthy, client‐owned dogs screened for eligibility to participate in the Vaccination Against Canine Cancer Study.

**Methods**: Dogs between ages of 5.5 and 11.5 years and mixed breed or breeds at higher risk for cancer development were evaluated at three study sites. Physical examination with aspiration of dermal and subcutaneous masses, CBC, biochemical profile, urinalysis, 3‐view thoracic radiographs, and abdominal ultrasound were performed to identify occult cancer in all patients prior to study enrollment.

**Results**: Twenty‐three dogs were diagnosed with cancer, with another 11 dogs highly suspicious for cancer but not definitively confirmed, resulting in a 3.7% prevalence of undiagnosed cancer in this population of dogs. Twenty‐two (65%) were diagnosed by physical examination through aspiration of cutaneous or subcutaneous masses, with mast cell tumors diagnosed most commonly (n = 10).

**Conclusions and Clinical Importance**: The frequency of cancer diagnosis in this population of middle‐aged to older higher risk breeds is low, however, routine physical examination was able to detect the majority of these malignant tumors.

## SMALL ANIMAL INTERNAL MEDICINE

## Evaluation of histology, immunohistochemistry, clonality and STAT5B mutation detection in cats with chronic enteropathy

### **Anne C. Avery** ^1^; Emily Rout^2^, DVM, PhD, DACVP; Robert Burnett^3^, PhD; Sydney Bork^4^, DVM; Kelly Hughes^2^, DVM, PhD, DACVP; Lauren Harris^5^, DVM, PhD, DACVP; Paula Schaffer^2^, DVM, MS, DACVP; Craig Webb^6^, DVM, PhD, DACVIM (SAIM)

#### ^1^Colorado State University, Fort Collins, CO, USA; ^2^Assistant Professor, Microbiology, Immunology, Pathology, Colorado State University; ^3^Microbiology, Immunology, Pathology, Colorado State University, Fort Collins, CO, USA; ^4^Clinical Pathology Resident, Microbiology, Immunology, Pathology, Colorado State University, Fort Collins, CO, USA; ^5^Anatomic Pathologist, Microbiology, Immunology, Pathology, Colorado State University, Fort Collins, CO, USA; ^6^Professor, Clinical Sciences, Colorado State University, Fort Collins, CO, USA

**Background**: Differentiation of feline enteritis and intestinal small cell T‐cell lymphoma (SCL) by histology can be difficult and often relies on immunohistochemistry (IHC) and clonality testing. An activating mutation in STAT5B was described in feline SCL.

**Hypothesis/Objectives**: Hypothesis: histologic diagnosis of SCL varies across pathologists and detection of clonality and STAT5B mutation will aid in diagnosis. Objective: compare histology, CD3/CD20 IHC, clonality and STAT5B mutation results in feline intestinal biopsies.

**Animals**: Sixty‐six endoscopic duodenal and ileal biopsies from 41 client‐owned cats seen at a tertiary hospital with chronic enteropathy, which failed a 2‐week diet trial and did not have significant concurrent disease.

**Methods**: Three pathologists blindly categorized biopsy samples as enteritis, suspicious for SCL or definitive SCL, clonality was assessed by PCR for antigen receptor rearrangements, and detection of a STAT5B mutation was assessed by droplet‐digital PCR (cohort study).

**Results**: All pathologists agreed on the diagnosis in 41% of samples by histology. Twenty‐eight of 66 (42%) samples were diagnosed with SCL by at least 1 pathologist via histology; of these, 93% had a clonal T‐cell receptor rearrangement and 79% had mutant STAT5B detected. Among 14 samples diagnosed as enteritis histologically by all pathologists, 9/14 had suspicion for SCL following IHC, 5/14 had T‐cell clonality, and 2/14 carried mutant STAT5B.

**Conclusions and Clinical Importance**: Histologic diagnosis was often discordant across pathologists. Clonality and STAT5B mutation were detected in cats with suspicion but not definitive diagnosis of SCL. Cats with SCL frequently carry the STAT5B mutation and detection may be a useful ancillary test.

## Microbial indole catabolites of tryptophan in cats with chronic enteropathy

### **Patrick C. Barko** ^1^; Sina Marsilio^2^, DrMedVet, PhD, DACVIM (SAIM), DECVIM‐CA; Yu‐An Wu^3^, DVM; Arnon Gal^4^, DVM, MSc, PhD, DACVIM, DACVP; Gary Norsworthy^5^, DVM, DABVP (Feline); J. Scot Estep^6^, DVM DACVP; Jan Suchodolski^7^, MedVet, DrVetMed, PhD, AGAF, DACVM; Joerg Steiner^8^, MedVet, DrMedVet, PhD, DACVIM, DECVIM‐CA, AGAF; David Williams^9^, MA, VetMB, PhD, DACVIM, DECVIM

#### ^1^University of Illinois, Champaign, IL, USA; ^2^Assistant Professor, Veterinary Medicine and Epidemiology, School of Veterinary Medicine, University of California‐Davis, Davis, CA, USA; ^3^Graduate Research Assistant, PhD Student, Gastrointestinal Laboratory; School of Veterinary Medicine and Biomedical Sciences; Texas A&M University, Collage Station, TX, USA; ^4^Assistant Professor, Veterinary Clinical Medicine, College of Veterinary Medicine, University of Illinois, Champaign, IL, USA; ^5^Alamo Feline Health Center, San Antonio, TX, USA; ^6^Texas Veterinary Pathology, Spring Branch, TX, USA; ^7^Associate Professor, Gastrointestinal Laboratory; School of Veterinary Medicine and Biomedical Sciences; Texas A&M University, Collage Station, TX, USA; ^8^Professor, Gastrointestinal Laboratory; School of Veterinary Medicine and Biomedical Sciences; Texas A&M University, Collage Station, TX, USA; ^9^Professor, Veterinary Clinical Medicine, College of Veterinary Medicine, University of Illinois, Champaign, IL, USA

**Background**: Inflammatory bowel disease (IBD) and alimentary small cell lymphoma (SCL) are common chronic enteropathies (CE) in cats. Enteric microbiota dysbiosis is implicated in the pathogenesis of CE, but mechanisms of host‐microbiome interactions are poorly understood. Microbial indole catabolites of tryptophan (MICT) are gut microbial metabolites that participate in regulating mucosal barrier function and inflammation in humans and rodents. Previous metabolomic studies identified altered microbial tryptophan metabolism in cats with CE.

**Hypothesis/Objectives**: Quantify MICTs in sera of cats with IBD or SCL and compare them to healthy controls. We hypothesized that MICT concentrations would be decreased in cats with IBD or SCL compared with healthy controls.

**Animals**: Archived serum from cats with histopathological diagnoses of IBD (n = 44) or SCL (n = 31), and healthy controls (n = 51).

**Methods**: Quantitative LC‐MS was used to measure serum concentrations of tryptophan and seven different MICT metabolites.

**Results**: Serum concentrations of tryptophan, indolepropionate, indoleacrylate, indolelactate, indolepyruvate, and indolealdehyde varied significantly (Kruskal‐Wallis *P* < .05) among groups and were significantly (post‐hoc Dunn *P*_adj_ <.05) decreased in the IBD and SCL groups compared to healthy controls. Indolelactate concentrations were significantly decreased in the sera of cats with SCL compared to IBD (*P*_adj_ = .01), but no other analytes differed significantly between these groups.

**Conclusions and Importance**: Consistent with studies in humans and laboratory animals, our findings suggest microbial catabolism of tryptophan is decreased in cats with CE. MICTs are promising biomarkers that can be used to investigate the pathophysiologic consequences of dysbiosis in cats with IBD and SCL.

**Figure:**
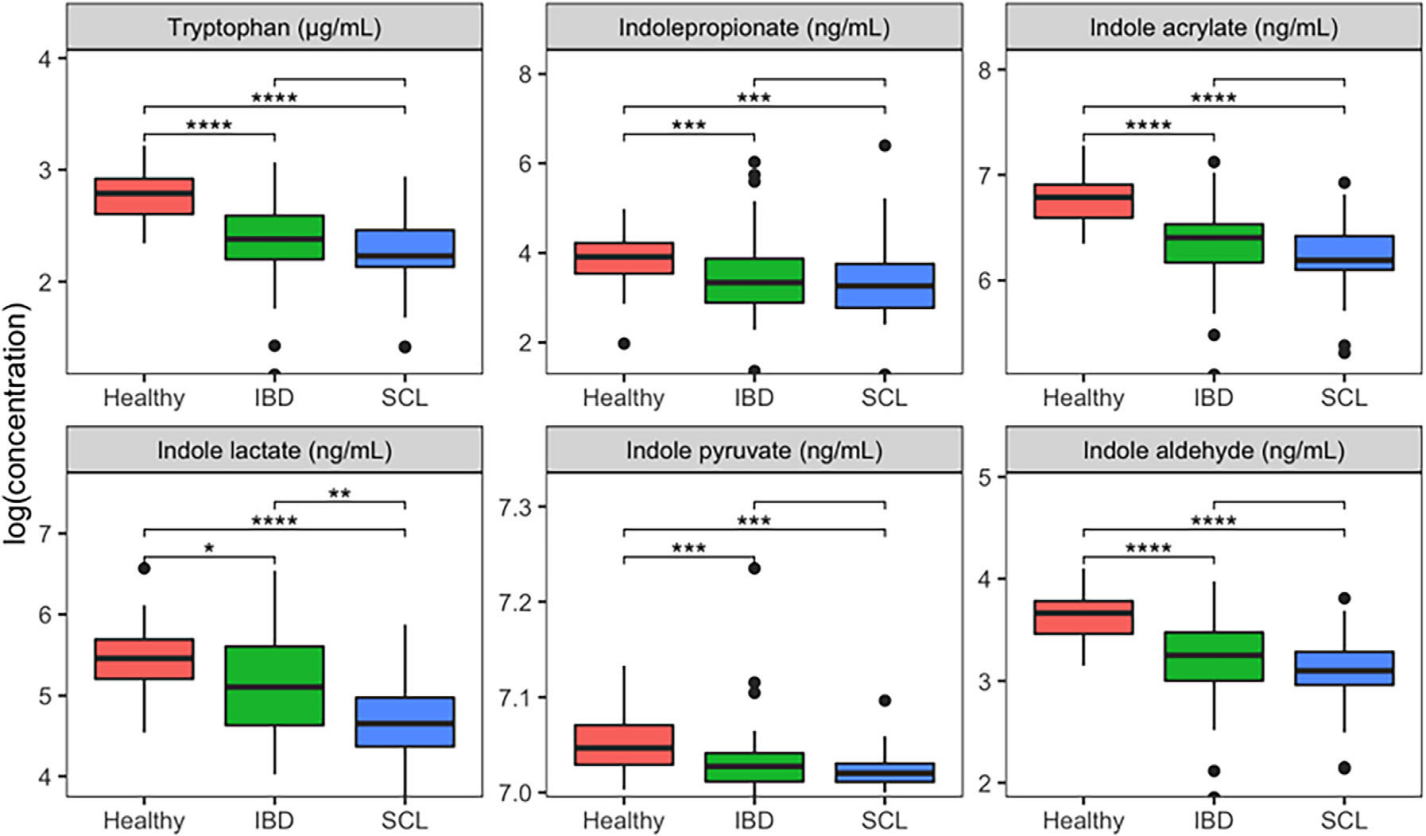
FIGURE 1. Serum microbial indole catabolites of tryptophan in cats with chronic enteropathies. Boxplots of analytes that varied significantly among cats with inflammatory bowel disease (IBD), alimentary small cell lymphoma (SCL), and healthy controls (Kruskal‐Wallis *P* < .05). Brackets show post‐hoc pairwise Dunn's tests comparing groups. Asterisks above brackets reveal statistically significant comparisons. **** *P* < .0001; *** *P* < .001; ** *P* < .01; * *P* < .05.

## Velagliflozin, an SGLT2 inhibitor, as once‐daily, oral solution, stand‐alone therapy for feline diabetes mellitus

### **Ellen N. Behrend** ^1^; Cindi Ward^2^, VMD, PhD, DACVIM; Victor Chukwu^3^, MD, DrPH; Audrey Cook^4^; Carla Kroh^5^; Patty Lathan^6^; Thomas Schermerhorn^7^; J. Catharine Scott‐Moncrieff^8^ ; Rebecca Voth^9^

#### ^1^Veterinary Information Network, Davis, CA, USA; ^2^Professor Emerita, Clinical Studies, University of Georgia, Athens, GA, USA; ^3^Biostatistican, Research and Development, Boehringer Ingelheim, Atlanta, GA, USA; ^4^School of Veterinary Medicine, Texas A&M, College Station, TX, USA; ^5^Boehringer Ingelheim Vetmedica GmbH; ^6^College of Veterinary Medicine, Mississippi State University, Mississippi State, MS, USA; ^7^College of Veterinary Medicine, Kansas State University, Manhattan, KS, USA; ^8^Purdue University, West Lafayette, IN, USA; ^9^Boehringer Ingelheim Animal Health USA Inc.

**Background**: A mainstay treatment for human diabetics, sodium‐glucose cotransporter‐2 (SGLT2) inhibitors hold promise for diabetic cats.

**Hypothesis/Objectives**: (a) Glycemic and clinical parameters will improve in >70% of diabetic cats receiving once‐daily velagliflozin. (b) <15% of cats will develop ketoacidosis.

**Animals**: Two hundred fifty‐two client‐owned diabetic cats; 214 (85%) naive diabetics (ND) and 38 (15%) insulin‐treated (IT).

**Methods**: Prospective trial evaluating once daily, oral velagliflozin. Physical examinations and blood collections performed days 0, 3, 7, 30, 60, 120, and 180. Data are median (range).

**Results**: At screening, blood glucose (BG) was 436 mg/dL (272‐676). At days 30, 60, 120, and 180, single BG after receiving velagliflozin was 153 (62‐480), 134 (64‐414), 128 (55‐461), and 125 (77‐384), respectively. Fructosamine at screening was 538 μmol/L (375‐794). On the same recheck days, fructosamine was 310 (204‐609), 286 (175‐531), 269 (189‐575), and 263 (203‐620). At day 180, 81% of cats had a BG and/or fructosamine within reference ranges; 88.6% (124/140) and 87.7% (121/138) showed improvement in polyuria and polydipsia, respectively. Eighteen cats (7.1%) developed ketoacidosis. Ketoacidosis was less common in ND (11/214; 5.1%) compared to IT cats (7/38; 18.4%). At ketoacidosis diagnosis, 14 cats (77.8%) had euglycemic ketoacidosis, that is, BG <250 mg/dL. Most ketoacidotic events (13/18; 72.2%) occurred within the first 7 days of treatment. No clinical hypoglycemia occurred.

**Conclusions and Clinical Importance**: Velagliflozin is effective as a stand‐alone oral solution therapy in feline diabetics with a low overall incidence of ketoacidosis.

## Correlation between urine anion gap and urine ammonia‐creatinine ratio in healthy and kidney disease cats

### **Alyssa Berman** ^1^; Andrew Specht^2^, DVM, DACVIM (SAIM); Shir Gilor^3^, DVM, MS, DACVP; Rebeca Castro^4^, BS; Kirsten Cooke^5^, DVM, DACVIM (SAIM); Autumn Harris^6^, DVM, DACVIM (SAIM)

#### ^1^University of Florida, Gainesville, FL, USA; ^2^Clinical Professor, Small Animal Clinical Sciences, University of Florida, Gainesville, FL, USA; ^3^Director, Clinical Pathology Service, Comparative, Diagnostic, and Population Medicine, University of Florida, Gainesville, FL, USA; ^4^Biological Scientist II, Small Animal Clinical Sciences, University of Florida, Gainesville, FL, USA; Clinical Associate Professor, Small Animal Clinical Sciences, University of Florida, Gainesville, FL, USA; ^6^Assistant Professor, Small Animal Clinical Sciences, University of Florida, Gainesville, FL, USA

**Background**: Ammonium excretion decreases as kidney function declines in several species including cats, and may have predictive or prognostic significance related to chronic kidney disease (CKD). Urine ammonia measurement is not readily available in clinical practice, so urine anion gap (UAG) has been proposed as a surrogate test.

**Objectives**: The primary aim was to determine degree of correlation between urine ammonia to creatinine ratio (UACR) and UAG in healthy cats and CKD cats. A secondary aim was to evaluate if there was a significant difference between the UAG of healthy and CKD cats.

**Animals**: Previously collected urine samples from client‐owned cats assessed to be healthy (n = 59) or to have stable CKD (n = 17) were thawed and processed.

**Methods**: Urine electrolytes were measured using a commercial chemistry analyzer. UAG was calculated as sodium + potassium − chloride. Urine ammonia and creatinine were previously measured using commercially available enzymatic assays and used to calculate UACR. Coefficient of determination (square of Pearson correlation coefficient) between UAG and UACR was calculated for both groups. Unpaired Student's *T*‐test was used to compare mean UAG of healthy vs CKD cats (*P* < .05).

**Results**: UAG had a very weak correlation with UACR in healthy (*R* ^2^ = .18) and CKD (*R* ^2^ = .07) cats. There was a significant difference between UAG in healthy and CKD cats (*P* < .002).

**Conclusions and Clinical Importance**: This calculation for UAG cannot be used as a substitute for UACR in cats. The clinical significance of the difference between healthy and CKD cats remains unknown.

## Effects of preservative and volume on diagnostic parameters for urine samples from dogs and cats

### **Kayla Dunn**; Dennis Chmiel, DVM, MBA; Becky Gallant, BS, CVT; Parker Jackson, MSc; Matthew Krecic, DVM, MS, MBA, DACVIM

#### MySimplePetLab

**Background**: The effect of urine preservative on select biochemical and microscopic parameters for dog urine samples was shown in a previous report at 7 mL urine per preservative tube. Collection of 7 mL of urine is not always possible from clinic or from home.

**Objective**: Objectives were to compare diagnostic quality of 1 mL (lower volume) urine samples versus 7 mL (manufacturer recommended) urine samples stored at room temperature in BD Vacutainer preservative tubes analyzed on day zero and day three (D0 and D3).

**Animals**: Nine pooled dog and four pooled cat urine samples (n = 13) were collected from a veterinary clinic.

**Methods**: Samples were separated into four groups (D0‐1 mL, D3‐1 mL, D0‐7 mL, D3‐7 mL). Investigators used refractometer for specific gravity, Zoetis VETSCAN UA Urine Analyzer for biochemical parameters, and Heska's Element AIM™ for urine sediment evaluation and reported results with use of fixed quantitative (specific gravity) or semi‐quantitative scales (all biochemical and microscopic parameters).

**Results**: Results for red and white blood cells, epithelial cells, crystals, and analytes for blood and pH were consistent across groups, with minimal variance observed in specific gravity. Volume‐dependent variability (between 1 and 7 mL) was noted in protein analytes for four dog and three cat samples but not across days (D0 to D3). Data were insufficient to assess ketones, bilirubin, glucose, and casts.

**Conclusion**: Protein variability may be explainable by manufacturer's notes although this was not consistent across all samples. Further studies will compare this urine protein variability with urine protein/creatinine ratio and microalbumin concentration to help assess clinical relevance.

## Use of sodium zirconium cyclosilicate for the management of hyperkalemia in dogs and cats

### **J.D. Foster**

#### Friendship Hospital for Animals, Washington, DC, USA

**Background**: Hyperkalemia is a life‐threatening emergency requiring prompt correction. Acute kidney injury and chronic kidney disease patients may experience hyperkalemia. Therapies that correct hyperkalemia through kaluresis may be less effective in these patients. Sodium zirconium cyclosilicate (SZC) is approved for managing hyperkalemia in humans, by exchanging potassium for sodium across the gut capillaries.

**Hypothesis/Objectives**: To evaluate the efficacy of SZC in dogs and cats with naturally occurring hyperkalemia.

**Animals**: Dogs and cats with hyperkalemic kidney disease.

**Methods**: Retrospective medical record review identified patients who were administered SZC and had at least one recheck assessment of hyperkalemia. Patients who received other therapies to mitigate hyperkalemia were excluded from analysis.

**Results**: Nine dogs and 2 cats were eligible for enrollment. Two dogs were administered SZC to control interdialytic hyperkalemia. The median starting serum potassium concentration was 7.0 mmol/L (range 4.6‐6.6 mmol/L). The median dose of SZC was 0.11 g/kg (range 0.08‐0.19 g/kg). Most received this dose PO q 8 hours for the first 24 hours, then q24h thereafter. Median time to recheck potassium as 20 hours (range 1‐288 hours). The serum potassium decreased in 5/7 (71.4%) dialysis independent patients. Median change in potassium was −1.31 mmol/L (range −2.1 to 1.8 mmol/L). Dose of SZC in the two patients who experienced progressive hyperkalemia was 0.08 g/kg, where all other patients received >0.11 g/kg. The two dialysis patients had resolution of their interdialytic hyperkalemia following initiation of SZC.

**Conclusions and Clinical Importance**: SZC is an effective therapy for the management of hyperkalemia. Further study is needed to optimize effective dose.

## Characterizing the intrinsic nervous system of gallbladders from normal dogs and dogs with mucocele formation

### **Kaitlin V. Daly**; Jody Gookin; Elyse Wood; Gregory Bacola; Stephen Stauffer; Chloe Mariant; Laurianne Van Landeghem

#### North Carolina State University, Raleigh, NC, USA

**Background**: Pathogenesis of canine gallbladder mucocele formation is poorly understood. Hallmark features are secretion of abnormal mucus by the epithelium, poor contractility, and vascular infarction. Secretion, contractility, and vasomotor tone are collectively influenced by the intrinsic nervous system of the gallbladder. Our understanding of the local nervous system of the canine gallbladder is limited.

**Objective**: To characterize the neuronal and glial network of healthy and mucocele gallbladders for differences plausibly contributory to mucocele pathogenesis.

**Samples**: Gallbladders from apparently healthy dogs and dogs with mucocele formation.

**Methods**: Immunofluorescence imaging was used to characterize the neuronal and glial network of healthy and mucocele gallbladders. Cryosections and full‐thickness samples of solvent‐cleared intact gallbladders (iDISCO) were treated with antibodies specific for neurons (tubulin β3; Tuj), glia (glial fibrillary acidic protein, GFAP) and epithelium (EpCAM CD326). Cryosections were imaged using traditional fluorescence microscopy and full‐thickness specimens by light‐sheet microscopy.

**Results**: Healthy canine gallbladders contained an extensive neuronal and associated glial network consisting of neuronal projections to the epithelium, muscularis, and perivascular. Ganglia were observed in both the lamina propria and at the interface of the muscularis and adventitia. In mucocele gallbladders neuronal projections and ganglia were largely absent, while the glial network remained intact but with reduced density.

**Conclusions**: Canine gallbladder mucocele formation is associated with a striking loss of neurons, ganglia, and neuronal projections with relative preservation of glia. While the mechanism(s) responsible for loss of neurons are unknown, their absence undoubtedly contributes to abnormal gallbladder secretory, motor, and possibly vasomotor function.

## Tracheobronchial lymphadenopathy in dogs with pulmonary coccidioidomycosis

### **Jared A. Jaffey** ^1^; Rebecca Urion^2^, DVM, MAg, DACVR‐DI, DACVR‐EDI; Eric Hostnik^3^, DVM, MS, DACVR, DACVR‐EDI; George Moore^4^, DVM, PhD, DACVIM (SAIM), DACVPM (Epi); Andrew Hanzlicek^5^, DVM, MS, DACVIM (SAIM)

#### ^1^College of Veterinary Medicine, Midwestern University, Glendale, AZ, USA; ^2^Assistant Professor, Veterinary Clinical Sciences, The Ohio State University, Colombus, OH, USA; ^3^Associate Professor, Veterinary Clinical Sciences, The Ohio State University, Colombus, OH, USA; ^4^Associate Professor, Purdue University, West Lafayette, IN, USA; ^5^Director, Veterinary Medicine & Research, MiraVista Veterinary Diagnostics, Indianapolis, IN, USA

**Background**: Tracheobronchial lymphadenopathy (TBL) is a common radiographic finding in dogs with pulmonary coccidioidomycosis. The time‐to‐resolution and predictive variables of this clinical finding remain unexplored in dogs.

**Hypothesis/Objectives**: To determine whether any baseline variables are associated with time‐to‐resolution of TBL after initiation of treatment with fluconazole.

**Animals**: Thirty‐two client‐owned dogs with newly diagnosed pulmonary coccidioidomycosis.

**Methods**: Prospective cohort study. Thoracic radiographs and other diagnostic tests were performed at baseline and once every 3 months after initiation of fluconazole administration until remission or for a maximum of 12 months. Severity of radiographic TBL was evaluated with a subjective and semi‐objective classification scheme.

**Results**: Twenty‐five (78%) dogs had TBL present on baseline radiographs. Resolution of TBL occurred in most dogs within 3 (72%, 18/25) to 6 months (80%, 20/25) of starting fluconazole administration. Only 1 (4%) dog failed to have TBL resolve within the study period. The median time‐to‐resolution of TBL was 96 days (range, 72‐386 days). Cox's proportional hazard univariate analysis identified increasing TBL severity (hazard ratio [HR] = 0.40, 95% CI: 0.19‐0.84, *P* = .02) and semi‐objective size, length:T4 vertebral body ratio (HR = 0.41, 95% CI: 0.20‐0.82, *P* = .01) as variables associated with longer time‐to‐resolution.

**Conclusions and Clinical Importance**: Radiographic TBL should resolve in most dogs with pulmonary coccidioidomycosis within 3 to 6 months of initiating fluconazole administration. Dogs with larger radiographic tracheobronchial lymph nodes at the time of diagnosis have a higher likelihood of taking longer to resolve after initiating fluconazole treatment.

## Association of the novel benzimidazole resistance marker Q134H with F167Y in dogs with *Ancylostoma caninum*

### **Christian M. Leutenegger** ^1^; Abhinaya Venkatesan^2^; Christian Savard^1^; Michelle Evason^1^; Jennifer Willcox^1^; Cathy Meeks^1^; Cecilia Lozoya^1^; Jeffrey Tereski^1^; Samantha Loo^1^; Jan Andrews^1^; John Gilleard^2^

#### ^1^Antech Diagnostics, Inc.; ^2^University of Calgary, AB, Canada

**Background**: Three single nucleotide polymorphisms (SNP) in the highly conserved β‐tubulin gene were identified to confer benzimidazole resistance in nematodes. Recently, a novel fourth marker, Q134H, was associated with functional benzimidazole resistance.

**Objective**: The objectives were to validate a Q134H specific real‐time PCR (qPCR), to determine frequency for the Q134H mutation and association with the F167Y genetic marker, in dogs naturally infected with *Ancylostoma caninum*.

**Animals**: A group of 143 fecal samples positive for *Ancylostoma* spp. by routine zinc sulfate centrifugal flotation (ZCF) and confirmed by *Ancylostoma* spp. qPCR were collected. Subsequently, samples were screened for the presence of both the Q134H and F167Y resistance markers.

**Methods**: *Ancylostoma* spp. detected by ZCF were confirmed by qPCR. Benzimidazole resistance markers Q134H and F167Y were detected by allele‐specific qPCR. The Q134H mutation was confirmed using conventional Sanger sequencing and nanopore amplicon deep sequencing protocols.

**Results**: Canine hookworm benzimidazole resistance (F167Y or Q134H) was detected in 39/143 (27.3%) of the hookworm confirmed samples. The F167Y allele resistance marker was detected in 31/143 (21.7%) samples. Nineteen (13.3%, 19/143) samples were positive for both markers while 12/143 (8.4%) were positive only for F167Y and 8/143 (5.6%) were positive only for the Q134H marker.

**Conclusions and Clinical Importance**: Anthelmintic resistance in hookworms is an emerging concern in veterinary medicine. This study confirms the utility of the F167Y marker for benzimidazole resistance identification in *Ancylostoma* positive samples, and highlights the importance in expanding testing to incorporate the novel Q134H marker.

## Single cell RNA sequencing atlas of canine duodenal mucosa in chronic inflammatory enteropathy and health

### **Alison C. Manchester** ^1^; Dylan Ammons^2^; Steven Dow^3^, DVM, PhD, DACVIM (SAIM); Michael Lappin^4^, DVM, DACVIM (SAIM), PhD

#### ^1^Colorado State University, Fort Collins, CO, USA; ^2^DVM/PhD student, Center for Immune and Regenerative Medicine, Clinical Sciences, Colorado State University, Fort Collins, CO, USA; ^3^Professor, Center for Immune and Regenerative Medicine, Clinical Sciences, Colorado State University, Fort Collins, CO, USA; ^4^Professor, Clinical Sciences, Center for Companion Animal Studies, Colorado State University, Fort Collins, CO, USA

**Background**: Canine chronic inflammatory enteropathy (CIE) is a complex disorder that currently requires intestinal biopsy histopathology for diagnosis. However, results of this diagnostic fail to elucidate disease‐related molecular processes. Cutting edge molecular tools, such as single cell RNA sequencing (scRNA‐seq), enable unprecedented investigation of disease pathogenesis, with the potential to shed light on the cellular and transcriptomic changes driving CIE.

**Hypothesis/Objectives**: To use scRNA‐seq to define canine duodenal cellular constituents and investigate how CIE alters transcriptional programs.

**Animals**: Three healthy research colony Beagles and 3 client‐owned CIE dogs with chronic diarrhea.

**Methods**: Descriptive case‐control study. For each dog, 15 mucosal pinch biopsies were collected from the duodenum via upper GI endoscopy. Biopsies were digested in preparation for scRNA‐seq. Samples from all 6 dogs were integrated into one dataset, then unsupervised clustering was completed. CIE associated changes were identified by comparing relative abundances of individual cell subsets and transcriptomic profiles.

**Results**: Transcriptional profiling of 40 000 cells from the 6 dogs' duodenal biopsies highlighted the presence of 35 unique cell populations, with ~50% of cells being immune and the remainder epithelial or stromal. Evaluation of integrated dataset revealed substantial intra‐dog variability. Despite this, analysis suggested CIE has an impact on the transcriptional programs of both immune and non‐immune cells.

**Conclusions and Clinical Importance**: We present a high‐resolution dataset demonstrating the cellular heterogeneity in canine duodenal mucosa in health and CIE, offering a new vantage point to understand intestinal pathobiology.

## The effect of gabapentin on blood pressure in cats with and without chronic kidney disease

### **Jessica M. Quimby** ^1^; Sarah Jones^1^; Ashlie Saffire^2^, DVM, DABVP (Feline); Katelyn Brusach^1^; Kim Kurdziel^3^; Zach George^3^; Rene Paschall^4^, DVM; Turi Aarnes^1^

#### ^1^The Ohio State University, Columbus, OH, USA; ^2^Cats Only Veterinary Hospital, Columbus, OH, USA; ^3^Vet student, The Ohio State University, Columbus, OH, USA; ^4^Resident, The Ohio State University, Columbus, OH, USA

**Background**: Anecdotal evidence suggests that gabapentin may decrease blood pressure (BP) in cats.

**Objective**: Assess the effect of gabapentin on BP in cats with and without CKD.

**Animals**: Thirty client‐owned cats were enrolled: 14 cats with stable CKD (IRIS Stage 2 and 3) and 16 apparently healthy cats (serum creatinine <1.6 mg/dL and USG >1.035).

**Methods**: A randomized, blinded, placebo‐controlled crossover study was performed. Cats were evaluated twice, ~1 week apart, and BP (Doppler) was obtained 3 hours after receiving either a single oral dose of 10 mg/kg gabapentin or placebo. For each cat, BP readings were obtained at each visit using the same Doppler and sphygmomanometer unit, same cat holder and doppler operator, and in the same location.

**Results**: Administration of a single 10 mg/kg oral dose of gabapentin resulted in a significant decrease in BP (median 125 mm Hg, range 82‐170) when compared to placebo (151, 102‐191; *P* < .001). In the CKD subgroup, gabapentin resulted in a significant decrease in BP (137 mm Hg, 96‐170) when compared to placebo (157, 102‐191; *P* = .003). In the healthy cat subgroup, gabapentin resulted in a significant decrease in BP (121 mm Hg, 82‐139) when compared to placebo (137, 102‐177; *P* = .002). Median change in BP was 12 mm Hg (−10 to 95) for healthy cats and 20 mm Hg (−21 to 43) for CKD cats (no significant difference between subgroups).

**Conclusions and Clinical Importance**: The effect of gabapentin on BP should be taken into consideration when cats receive gabapentin for visit‐associated stress.

## Itraconazole metabolite biotransformation by hepatic cytochrome P450 enzymes in the dog

### **Jennifer M. Reinhart** ^1^; Jennifer Applebaum^2^; Stephen Grisoli^2^; Zhong Li^3^

#### ^1^University of Illinois Urbana‐Champaign, Urbana, IL, USA; ^2^Veterinary Clinical Medicine, University of Illinois Urbana‐Champaign, Urbana, IL, USA; ^3^Duke Proteomics and Metabolomics Shared Resource, Duke University, Durham, NC, USA

**Background**: Itraconazole (ITZ) is a commonly used azole antifungal in dogs but has an unacceptable rate of treatment failure and adverse reactions. Understanding species‐specific metabolism of ITZ may be important in predicting clinical outcomes. ITZ undergoes serial metabolism by the hepatic cytochrome P450 (CYP) enzymes to hydroxy‐ITZ, keto‐ITZ, and N‐desalkyl‐ITZ. The first step is mediated by CYP2D15 and CYP3A12, but the specific isoforms responsible for the latter reactions are unknown.

**Hypothesis/Objectives**: Identify the CYP isoforms that mediate the hydroxy‐ITZ to keto‐ITZ and keto‐ITZ to N‐desalkyl‐ITZ reactions in dog liver.

**Animals**: None.

**Methods**: Reaction conditions were optimized by incubating substrates (hydroxy‐ITZ or keto‐ITZ) with dog liver microsomes and products (keto‐ITZ or N‐desalkyl‐ITZ) were quantified by LC‐MS. Substrates were incubated with recombinant canine CYP enzymes (CYP1A1, CYP1A2, CYP2B11, CYP2C21, CYP2C41, CYP2D15, CYP3A12, CYP3A26). Reaction velocities were compared between CYPs before and after correction for relative abundance of isoforms within dog liver microsomes. For CYPs with the highest corrected reaction velocities, activities for the respective reactions were confirmed using isoform‐specific inhibitors.

**Results**: CYP2D15 and CYP3A12 showed the highest uncorrected and corrected reaction velocities for both hydroxy‐ITZ to keto‐ITZ and keto‐ITZ to N‐desalkyl‐ITZ reactions. Quinidine and erythromycin selectively inhibited both reactions in recombinant CYP2D15 and CYP3A12, respectively.

**Conclusions and Clinical Importance**: CYP2D15 and CYP3A12 both appear to contribute to the second and third steps of ITZ metabolism in dog liver. Genetic or acquired factors affecting expression or function of these isoforms could impact clinical outcomes of this drug.

## Treatment with the sodium‐glucose cotransporter‐2 inhibitor bexagliflozin in cats newly diagnosed with diabetes mellitus

### **J. Catharine** **Scott‐Moncrieff** ^1^ ; Michael Hadd^2^; Stephen Bienhoff^3^; Susan Little^4^; Samuel Geller^5^; Jennifer Ogne‐Stevenson^3^ ; Thomas Dupree^6^

#### ^1^Purdue University, West Lafayette, IN, USA; ^2^IncreVet, Boston, MA, USA; ^3^Argenta, New Brunswick, NJ, USA; ^4^Bytown Animal Hospital, Ottawa, ON, Canada; ^5^Quakertown Veterinary Clinic, Quakertown, PA, USA; ^6^IncreVet, Boston, MA, USA

**Background**: Sodium‐glucose cotransporter‐2 (SGLT2) inhibitors improve glycemic control in human type 2 diabetes mellitus (DM) and have potential for treatment of feline DM.

**Hypothesis/Objectives**: The SGLT2 inhibitor, bexagliflozin, is safe and effective as a stand‐alone oral treatment for DM in naive diabetic cats.

**Animals**: Eighty‐four client‐owned cats with naive DM.

**Methods**: Historically controlled prospective clinical trial. Cats received 15 mg bexagliflozin orally once daily for 180 days. Efficacy was evaluated at day 56.

**Results**: Of 81 evaluable cats at day 56, at least one clinical sign of DM improved in 91% cats (Table 1) and quality of life improved in 87% cats. Improvements were generally maintained through day 180 of the study. Mean serum glucose decreased from 439.8 mg/dL (95% CI: 424.2‐455.4) on day 0, to 161.4 mg/dL (95% CI: 132.1‐161.5) on day 56, and 144.4 mg/dL (95% CI: 126.1‐162.6) on day 180. Mean serum fructosamine decreased from 542.5 μmol/L (95% CI: 523.0‐562.0) on day 0, to 301.3 μmol/L (95% CI: 280.4‐322.2) on day 56, and 305.6 μmol/L (95% CI: 269.1‐342.0) on day 180. Serum β‐hydroxybutyrate concentration decreased from 13.7 mg/dL (95% CI: 11.3‐16.1) at baseline to 2.91 mg/dL (95% CI: 1.8‐4.1) on day 56, and 3.21 mg/dL (95% CI: 1.3‐7.5) on day 180.

Serious adverse events occurred in 8 cats, with three deaths. Euglycemic diabetic ketoacidosis occurred in 4 cats of which one died.

**Conclusion and Clinical Importance**: Bexagliflozin reduced hyperglycemia and clinical signs in naive diabetic cats.

**Table:** TABLE 1. Owner assessment of clinical signs at day 56 compared to day 0.

Variable	Significantly improved		Somewhat improved		Unchanged		Worsened		Success	
	n	%	n	%	n	%	n	%	n	%
Polyphagia	26	34.67%	21	28.00%	24	32.00%	4	5.33%	47	62.67%
Polydipsia	33	44.00%	28	37.33%	12	16.00%	2	2.67%	61	81.33%
Polyuria	29	38.67%	27	36.00%	17	22.67%	2	2.67%	56	74.67%

## Blood pressure in hyperthyroid cats before and after radioiodine treatment

### **Lisa Stammeleer** ^1^; Pilar Xifra^2^, DVM; Sara Serrano^2^; Sylvie Daminet^1^; Ellen Vanden Broecke^1^; Mark Rishniw^3^; Mark Peterson^4^, DACVIM

#### ^1^Ghent University, Flanders, Belgium; ^2^Iodocat, Leganés, Spain; ^3^Cornell University, Ithaca, NY, USA; ^4^Director, Animal Endocrine Clinic, New York, NY, USA

**Background**: Feline hyperthyroidism is commonly associated with systemic hypertension, with reported prevalence of 9% to 48%. Although hypertension might be expected to resolve once methimazole restores euthyroidism, it can persist or only first develop after treatment. Further, the effect of radioiodine treatment or iatrogenic hypothyroidism on resolution or development of hypertension has not been investigated.

**Objectives**: To determine proportion of hyperthyroid cats with hypertension (systolic blood pressure [SBP] >160 mm Hg); persistence or first development of hypertension after successful ^131^I treatment, and correlation of post‐treatment hypertension with azotemia or iatrogenic hypothyroidism.

**Animals**: A total of 407 hyperthyroid cats, 257 rechecked post‐treatment.

**Methods**: Hyperthyroid cats had SBP measured by Doppler; 257 had SBP rechecked 6 months after successful ^131^I treatment.

**Results**: Of untreated hyperthyroid cats, 111/407 (27%) and 88/257 (34%) were hypertensive. A higher proportion of hypertensive cats were nervous/excited compared with normotensive cats (45% vs 14%; *P* < .001). After ^131^I treatment, 44/257 (17%) previously hypertensive cats normalized SBP, whereas 44/257 remained hypertensive; 16 (6%) other cats first developed hypertension after treatment. Of these 60 cats, only 5 (8%) were azotemic and 6 (10%) were hypothyroid. A higher proportion of cats remaining hypertensive had nervous/excited demeanor than did normotensive cats (41% vs 19%; *P* = .002).

**Conclusions/Clinical Importance**: Hyperthyroid cats infrequently (27%) had SBP >160 mm Hg, half of which normalized after ^131^I treatment. Cats rarely developed post‐treatment hypertension. Persistent or newly diagnosed hypertension in these cats was unrelated to azotemia or iatrogenic hypothyroidism. Nervousness/anxiety in hypertensive cats suggests that many of these cats might have “white coat” situational hypertension.

## Evaluating acidic gastroesophageal reflux in french bulldogs with sliding hiatal herniation using Bravo pH monitoring

### **Tarini V. Ullal** ^1^; Stanley Marks^2^, BVSc, PhD, DACVIM (SAIM, Oncology), DACVN; Nuen‐Tsang Yang^1^; Sandra Taylor^1^

#### ^1^University of California‐Davis, Davis, CA, USA; ^2^Faculty Professor, Medicine and Epidemiology, University of California‐Davis, Davis, CA, USA

**Background**: Sliding hiatal herniation (SHH) and gastroesophageal reflux (GER) commonly occur in French bulldogs. Methods to quantify GER and the effect of brachycephalic obstructive airway syndrome (BOAS) surgery are lacking.

**Hypothesis/Objectives**: Implement a wireless pH capsule (Bravo Calibration‐free, Medtronic, MN) to measure acidic reflux in French bulldogs with SHH, pre‐ and post‐BOAS surgery.

**Animals**: Eleven French bulldogs videofluoroscopically diagnosed with SHH, off acid suppressant(s) for >2 weeks.

**Methods**: A pH capsule was endoscopically placed in the esophagus during a brief anesthesia. Up to 96 hours of data was acquired while the dog was awake and at home. The owner simultaneously logged clinical signs. In 4 dogs, pH monitoring was repeated 2 to 4 months following BOAS surgery. Spearman's correlation and Wilcoxon rank‐sum tests evaluated factors correlated with acid exposure time (AET).

**Results**: Medians (Q1‐Q3) for age and weight were 30 months (17‐35.5) and 10.3 kg (8.9‐12.2). BOAS severity was mild in 3, moderate in 3, and severe in 5 dogs. Medians (Q1‐Q3) for AET and number of reflux events were 5.8% (2.2‐7) and 114 (37‐188). BOAS severity (*P* = .08) and esophagitis (*P* = .9) were not correlated with AET, but age was negatively correlated (*P* = .03). Median probability that owners observed regurgitation during reflux events was 73%, range 0% to 99%. In 3 of 4 dogs that underwent BOAS surgery, AET improved following surgery (Table 1).

**Conclusions and Clinical Importance**: Wireless pH monitoring documented acidic GER in French bulldogs with SHH, captured subclinical events, and showed improvements following BOAS surgery.

**Table:** TABLE 1. Percent acid exposure time (AET) in 4 dogs that underwent surgical correction of brachycephalic obstructive airway syndrome (BOAS).

Dog	% AET	
	Pre‐BOAS surgery	Post‐BOAS surgery
1	1.3	0.6
2	2.7	1.7
3	5.8	5.1
4^a^	1.7	1.6

Abbreviations: % AET, percent acid exposure time; BOAS, brachycephalic obstructive airway syndrome.

^a^ Dog 4 underwent both BOAS and hiatal hernia surgery.

## Gastrointestinal adverse effects of ophthalmic nonsteroidal anti‐inflammatory drugs in dogs

### **Laura R. Van Vertloo** ^1^; Rachel Allbaugh^1^; Karin Allenspach^1^; Lionel Sebbag^2^; David Borts^1^; Jon Mochel^1^

#### Iowa State University, Ames, IA, USA

**Background**: While gastrointestinal (GI) ulceration is a well‐recognized adverse effect associated with systemic nonsteroidal anti‐inflammatory drugs (NSAIDs), the risk of systemic adverse effects from topical ophthalmic application of NSAIDs is unknown.

**Hypothesis/Objectives**: To investigate systemic concentrations and GI adverse effects of ophthalmic ketorolac 0.5% and diclofenac 0.1%.

**Animals**: Eleven healthy purpose‐bred adult beagle dogs.

**Methods**: Dogs were randomly assigned to receive either ketorolac (N = 6) or diclofenac (N = 5) one drop in both eyes four times daily for 28 days in a parallel design. Upper GI endoscopy was performed on Day 0 and Day 28 with mucosal lesion scores (0‐7) assigned to each region evaluated. Blood samples were collected 15 minutes following eyedrop administration on Day 14, 21, and 28 for drug quantification in plasma using high‐performance liquid chromatography/mass spectrometry.

**Results**: Erosions and/or ulcers developed in 4/6 ketorolac‐treated dogs and 1/5 diclofenac‐treated dogs. Post‐treatment mucosal lesion score for the antrum was higher in the ketorolac group than in the diclofenac group (*P* = .01) but not significantly different for any other region. Vomiting and decreased appetite events were observed uncommonly and were not significantly different between treatment groups. Ketorolac and diclofenac were detected in the plasma at all timepoints (ketorolac median 173.5‐191 ng/mL, diclofenac median 20.6‐27.5 ng/mL).

**Conclusions and Clinical Importance**: Ketorolac and diclofenac were absorbed systemically after ophthalmic administration and associated with the development of GI ulceration and erosion, with higher plasma concentrations and more severe GI lesions associated with ketorolac.

## Evaluation of serum galectin‐3 concentrations in healthy cats and in cats with ureteral obstruction

### **Dennis Woerde** ^1^; Carrie Palm^2^, DVM, MAS, DACVIM (SAIM); Krystle Reagan^3^, DVM, PhD, DACVIM (SAIM)

#### ^1^University of California‐Davis, Davis, CA, USA; ^2^Professor of Clinical Internal Medicine, University of California‐Davis, Davis, CA, USA; William Culp^4^, DVM, DACVS; ^3^Assistant Professor, Medicine & Epidemiology, University of California‐Davis, Davis, CA, USA; ^4^Professor, Surgical & Radiological Sciences, University of California‐Davis, Davis, CA, USA

**Background**: Early acute kidney injury (AKI) diagnosis is difficult with traditional biomarkers (eg, creatinine). Novel injury biomarkers may diagnose AKI secondary to ureteral obstruction (UO) earlier, before irreversible damage develops. Serum Galectin‐3 (sGal‐3), a potential novel biomarker, is a protein present in kidney tubules shown to increase in rats with UO.

**Hypothesis/Objectives**: To compare sGal‐3 in healthy cats and cats with UO.

**Animals**: Fifteen healthy control cats and 22 cats with UO. For control cats, creatinine and SDMA were within reference intervals and ultrasound showed minimal to no renal changes.

**Methods**: Serum was collected in all cats. A feline specific sGal‐3 enzyme‐linked immunosorbent assay (ELISA) was used to determine sGal‐3 concentrations. Samples were performed in duplicate, and data included if the coefficient of variation between samples was <20%. Shapiro‐Wilk testing was used to evaluate for normality and parametric statistics were performed. *P* < .05 was considered significant.

**Results**: Mean serum Gal‐3 was lower in healthy cats (274.3 pg/mL [±146.5 SD]) compared to cats with UO (707.7 pg/mL [±223.3 SD]) (*P* < .0001). There was no difference in sGal‐3 concentrations between cats with unilateral or bilateral UO (*P* = .2) and no correlation between Gal‐3 and body weight or age. With a cut‐off of 500 pg/mL, sGal‐3 had a sensitivity of 86% and specificity of 100% for UO diagnosis.

**Conclusions and Clinical Importance**: Given the high morbidity and mortality of UO in cats, early diagnosis is critical. Gal‐3 may be a potential biomarker for diagnosis of UO or other AKI causes in cats.

