# Erratum

**DOI:** 10.1111/jvim.16968

**Published:** 2023-12-18

Erratum for “2023 ACVIM Forum Research Report Program” https://doi.org/10.1111/jvim.16902

First published: 06 November 2023

This article corrects the following:

For the abstract, “Balloon Valvuloplasty for Pulmonary Valve Stenosis: Multicenter Collaborative Study Across Pediatric and Veterinary Cardiology Centers,” the credentials for Lauren Markovic credentials DVM, DACVIM (Cardiology).

